# Temporal expression profiling of long noncoding RNA and mRNA in the peripheral blood during porcine development

**DOI:** 10.5713/ajas.19.0313

**Published:** 2019-08-03

**Authors:** Yiren Gu, Rui Zhou, Long Jin, Xuan Tao, Zhijun Zhong, Xuemei Yang, Yan Liang, Yuekui Yang, Yan Wang, Xiaohui Chen, Jianjun Gong, Zhiping He, Mingzhou Li, Xuebin Lv

**Affiliations:** 1 Animal Breeding and Genetics Key Laboratory of Sichuan Province, Sichuan Animal Science Academy, Chengdu, Sichuan 610066, China; 2 Farm Animal Genetic Resources Exploration and Innovation Key Laboratory of Sichuan Province, Sichuan Agricultural University, Chengdu, Sichuan 611130, China

**Keywords:** Long Noncoding RNA, mRNA, Pig, Peripheral Blood, Immune System Development

## Abstract

**Objective:**

We investigated the temporal expression profiles of long noncoding RNA (lncRNA) and mRNA in the peripheral blood of pigs during development and identified the lncRNAs that are related to the blood-based immune system.

**Methods:**

Peripheral blood samples were obtained from the pigs at 0, 7, 28, and 180 days and 2 years of age. RNA sequencing was performed to survey the lncRNA and mRNA transcriptomes in the samples. Short time-series expression miner (STEM) was used to show temporal expression patterns in the mRNAs and lncRNAs. Gene ontology and Kyoto encyclopedia of genes and genomes analyses were performed to assess the genes’ biological relevance. To predict the functions of the identified lncRNAs, we extracted mRNAs that were nearby loci and highly correlated with the lncRNAs.

**Results:**

In total of 5,946 lncRNA and 12,354 mRNA transcripts were identified among the samples. STEM showed that most lncRNAs and mRNAs had similar temporal expression patterns during development, indicating the expressional correlation and functional relatedness between them. The five stages were divided into two classes: the suckling period and the late developmental stage. Most genes were expressed at low level during the suckling period, but at higher level during the late stages. Expression of several T-cell-related genes increased continuously during the suckling period, indicating that these genes are crucial for establishing the adaptive immune system in piglets at this stage. Notably, lncRNA *TCONS-00086451* may promote blood-based immune system development by upregulating nuclear factor of activated T-cells cytoplasmic 2 expression.

**Conclusion:**

This study provides a catalog of porcine peripheral blood-related lncRNAs and mRNAs and reveals the characteristics and temporal expression profiles of these lncRNAs and mRNAs during peripheral blood development from the newborn to adult stages in pigs.

## INTRODUCTION

A better understanding of porcine immune system development is needed because of the agricultural importance of pigs, the role of pigs as potential reservoir hosts for zoonotic diseases, and increasing awareness of the benefits of using pigs as preclinical models for human disease and therapy [1]. Previous research has reported that mammals experience a critical stage in development that extends from late gestation to weaning [2]. Piglets suffer high mortality and morbidity during this stage due to various diseases [3], likely because this stage is a critical window when the adaptive immune system is developing, and the temporary protection provided by passive immunity and pre-adaptive antibodies is being replaced [4]. Therefore, accurate knowledge of the immune system development after birth, which relies on strict regulation of gene expression profiles and their changes during development, is needed. Mammalian blood acts as a pipeline for the immune system. Assessing the abundance of transcripts in the blood offers a snapshot of the complex immune regulatory networks operating throughout the body [5]. Compared with other tissues, blood can be sampled repeatedly over time, and constitutes a key feature for monitoring the body’s immune status during development [5]. However, to our knowledge, the mRNA and long noncoding RNA (lncRNA) expression profiles in the peripheral blood of pigs during postnatal development have not been comprehensively analyzed.

The lncRNAs are a class of RNA transcripts longer than 200 nucleotides with little or no evidence of protein coding capacity [6]. Recent evidence has shown the importance of lncRNAs in regulating gene expression [7], and their roles in immune regulation have also become a research focus. lncRNAs are widely expressed in different immune cells and play various roles in immune cell development, differentiation and activation [8]. For example, the lncRNA p50-associated Cox2 extragenic RNA is involved in regulating of cyclooxygenase 2 gene expression and the nuclear factor kappa-B signaling pathway [9]. In humans, *lnc*-DC is essential for differentiating monocytes into dendritic cells [10]. Thus, lncRNA likely plays a significant role in regulating postnatal blood-based immune system development.

We hypothesized that postnatal blood development in pigs is closely associated with the changes in lncRNA and mRNA expression profiles over time. We performed RNA-sequencing (RNA-seq) analysis on peripheral blood from pigs at the age of 0 day (D0), 7 days (D7), 28 days (D28), 180 days (D180), and 2 years (Y2), which represent the newborn [11], suckling [12], weaning [12], sexual maturity [13], and adult [11] stage in pigs, respectively. These five important developmental stages represent five critical physiological periods during postnatal blood development in pigs. These time-course data are ideal for studying dynamic biological systems and identifying genes that are important in regulating blood-based immune system development. This study will contribute to a better comprehension of the immune system changes in pigs and will serve as a framework for further mechanistic studies of the roles of lncRNAs in the blood-based immune system.

## MATERIALS AND METHODS

### Animals and samples

Ten healthy female Qingyu pigs were divided into five time points (two replicates for each time point): D0 (just after birth), D7 (7 days old), D28 (28 days old), D180 (180 days old), and Y2 (2 years old). Peripheral blood samples were collected from each pig, immediately immersed into liquid nitrogen, and stored at −80°C until subsequent RNA isolation.

The Institutional Animal Care and Use Committee of the College of Animal Science and Technology, Sichuan Agricultural University, Sichuan, China, approved all animals and samples used in this study under permit No. DKY-S20174215.

### RNA isolation, library construction, and sequencing

Total RNA was extracted using TRIzol reagent (Life Technologies, Beijing, China) following the manufacturer’s instructions. RNA-seq complementary DNA (cDNA) libraries were generated with total RNA using the Ribo-Zero kit (Epicentre). RNA was sequenced per the manufacturer’s standard procedures. High-quality strand-specific libraries were sequenced on Illumina’s HiSeq X Ten platform. Collectively, all porcine RNA-seq data constituted more than 100 Gb of high-quality data, of which, the quality of 90% of the bases was ≥Q30 (Table 1). The data underlying this study were deposited into the National Center for Biotechnology Information’s Gene Expression Omnibus series, under accession number GSE123923.

### Messenger RNA expression analysis

Sequence reads were aligned to the pig reference genome (*Sus scrofa* 11.1 from Ensemble) using Hisat2 (v2.0.4). Stringtie (v.1.3.3) was applied to quantify mRNA expression and obtain fragments per kilobase per million mapped reads (FPKM). We considered a mRNA to be expressed if it had an expression value greater than 0.5 FPKM (FPKM >0.5) in at least one sample. Differentially expressed (DE) mRNAs between adjacent time points within the suckling period and the late phase of development were tested (D0 vs D7, D7 vs D28, and D180 vs Y2). We used an adjusted p value of ≤0.05 and |log_2_ (fold change)| ≥1.5 as the cut-offs for statistical significance.

### LncRNA identification and expression analysis

To identify lncRNA, Stringtie (v.2.2.1) was used to assemble the mapped reads of each sample. Cuffmerge (part of Cufflinks v2.2.1) was used to merge the assembled transcripts. The transcripts that were annotated in the reference (marked by ‘c’ for partial matches and ‘=’ for full matches) were removed using Coffcompare (part of Cufflinks, v.2.2.1). Next, transcripts longer than 200 nt were retained to predict coding potential using a previously described approach [14]. We considered a lncRNA transcript to be expressed if it had an expression value greater than 0.1 FPKM (FPKM >0.1) in at least one sample. Supplementary Figure S1 shows the lncRNA identification steps. We merged the reference annotated gtf file and the lncRNA gtf file, which was extracted from previous steps as a new reference annotated gtf file. DE lncRNAs between adjacent time points within the suckling period and the late developmental phase were tested (D0 vs D7, D7 vs D28, and D180 vs Y2) using Cuffquant and Cuffdiff. We used an adjusted p value of ≤0.05 and |log_2_ (fold change)| ≥1.5 as cut-offs for statistical significance.

### Short time-series expression miner analysis

The short time-series expression miner (STEM) [15] clustering algorithm was performed to identify temporal gene expression profiles during blood development. Significantly enriched model profiles are indicated by different colors (Benjamini-adjusted p value<0.05).

### Functional enrichment analysis

We identified highly associated mRNAs for lncRNAs to predict their function. First, we collected nearby mRNAs within 100 kb upstream and downstream of the lncRNAs. Next, Hmisc was used to calculate Pearson correlations between the lncRNAs and the protein-coding genes. Highly correlated protein-coding genes (|r|>0.95 and p<0.05) were extracted. These protein-coding genes were defined as potentially highly related genes with similar structure or function to the lncRNAs.

The DE protein-coding genes and the highly associated genes for the lncRNAs were categorized into functional classes using the gene ontology (GO) terms and the Kyoto encyclopedia of genes and genomes (KEGG) pathways, respectively, using the DAVID webserver [16]. Only terms with Benjamini-adjusted p value≤0.05 were considered significant.

### Quantitative real-time polymerase chain reaction validation

Primers for five DE genes and internal control genes (Supplementary Table S1) were designed using Primer Premier 5.0 software with the optimal polymerase chain reaction validation (PCR) product length set between 100 and 300 bp and checked with Primer-BLAST (http://www.ncbi.nlm.nih.gov/tools/primer-blast/), then synthesized by BGI-Shenzhen (BGI-Shenzhen Co., Shenzhen, China). Following the manufacturer’s protocol, cDNA was synthesized using a PrimeScript 1st Strand cDNA Synthesis kit (Takara Biotechnology Co., Dalian, China). Quantitative real-time PCR (qRT-PCR) was performed as per the SYBR Premix Ex Taq II instructions (Takara Biotechnology Co., China) using a CFX96 real-time PCR detection system (Bio-Rad Co., Hercules, CA, USA).

The qRT-PCR measurements were performed with two replicates sample. The DE gene expression levels were normalized to β-actin. Relative gene expression levels were calculated by the 2^−ΔΔCt^ method, and data were expressed as the least square mean±standard error of the mean.

## RESULTS

### Sequencing results and quality control

To investigate the dynamic expression changes in lncRNAs and mRNAs in porcine peripheral blood during development, we performed high-throughput sequencing to survey the lncRNA and mRNA transcriptomes using porcine peripheral blood samples at five developmental time points (0, 7, 28, and 180 days and 2 years of age). Ten cDNA libraries were constructed and sequenced using the Illumina HiSeq X Ten platform and 150-bp paired-end reads were generated. A total of 857,831,714 raw reads were generated in all ten libraries (Table 1). After discarding adaptor sequences and low-quality reads, we obtained 831,081,192 clean reads. The percentage of clean reads in each library ranged from 95.80% to 97.59%. Using Hisat2, approximately 94.56% to 97.84% of the clean reads in each library could be mapped to the pig genome (*Sus scrofa* 11.1).

### Messenger RNA and lncRNA expression profiles in porcine peripheral blood

**Figure 1:**
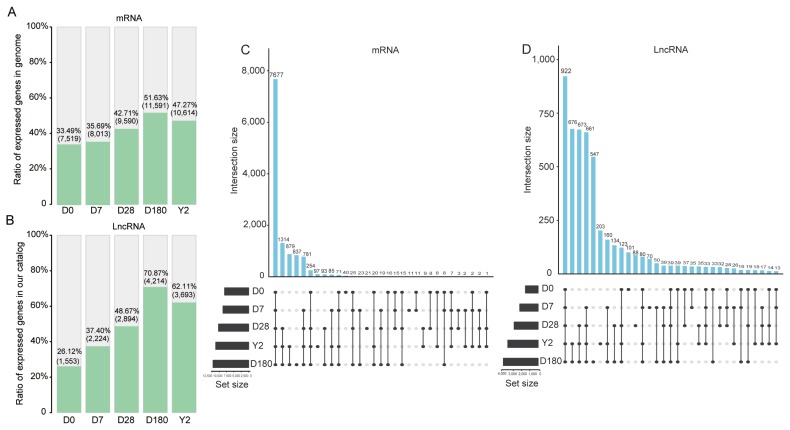
Gene expression characteristics at each time point. (A) Ratio of mRNAs expressed at the five time points in the genome. (B) Ratio of lncRNAs expressed at the five time points in our catalog. UpSet plot of the intersection of (C) mRNAs and (D) lncRNAs expressed at the five time points. lncRNAs, long noncoding RNA.

**Figure 2:**
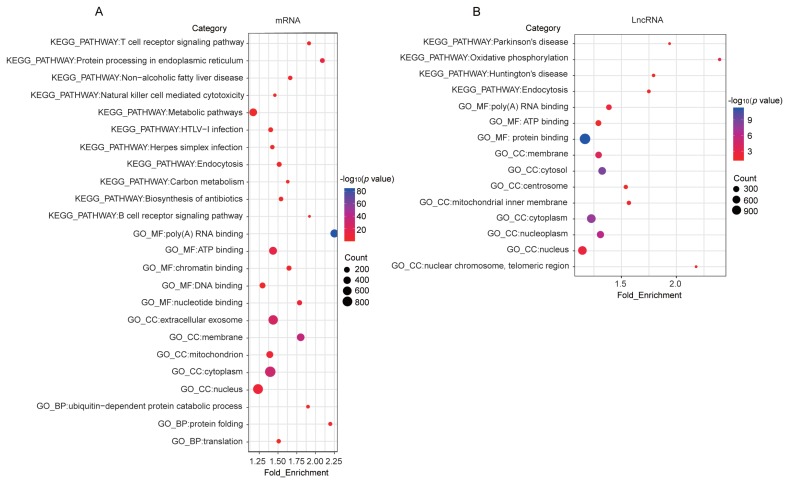
Functional categories of the commonly expressed genes identified in all five time points. The main enriched categories for (A) mRNAs and (B) long noncoding RNA (lncRNAs) commonly expressed in the five developmental time points. The function enrichment analysis of the genes was performed using DAVID and the listed terms were with Benjamini-corrected p value<0.05.

From the samples, 12,354 mRNA transcripts were expressed (FPKM >0.5 in at least one sample), and 5,946 lncRNA transcripts were substantially expressed (FPKM >0.1 in at least one sample). The lncRNAs shared similar features, such as relatively low exon numbers, low expression levels, short lengths, and low coding potentials, with previously reported vertebrate lncRNAs [14] (Supplementary Figure S2). Further analysis revealed that the number of expressed genes peaked at 180 days among the five time points. In detail, approximately 52% of the mRNA transcripts encoded in the genome and approximately 71% of the lncRNA transcripts encoded in our catalog were expressed at 180 days (Figure 1A, B). Comparing the expressed transcripts in every stage revealed a core group of 7,677 mRNA transcripts and 922 lncRNA transcripts in all five stages (Figure 1C, D). These genes may include the minimal set of genes required for the basic function for the pig blood. KEGG pathway and GO term annotations indicated that these mRNAs were mainly enriched in categories related to cellular component, immune response, genetic information processing, process of metabolism, and diseases (Benjamini-corrected p<0.05; Figure 2A). To investigate the possible functions of these lncRNAs, we searched for mRNAs 100 kb upstream and downstream of these lncRNAs. Nine hundred twenty-two lncRNAs were transcribed near 2,773 protein-coding neighbors (100 kb up- and downstream). Functional analysis showed that these lncRNAs were associated with regulating of cellular components, oxidative phosphorylation, and poly (A) RNA binding (Figure 2B). Several functions were commonly enriched for both universally expressed mRNAs and lncRNAs in all five time points, such as protein folding, nucleus, cytoplasm, ATP binding, poly(A) RNA binding and endocytosis. This suggested that maintaining of basic bood functions required co-regulation of mRNAs and lncRNAs, and lncRNAs may regulate mRNAs with similar biological functions [14].

**Figure 3:**
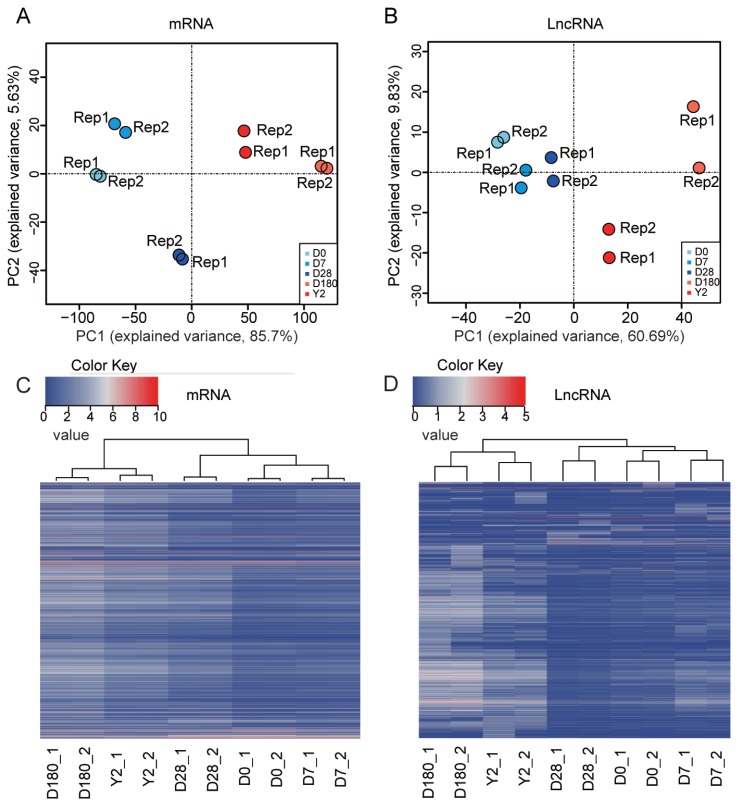
Global gene expression profiles of porcine peripheral blood during development. Principal component analysis (PCA) plot of (A) mRNAs and (B) lncRNAs performed based on log2-transformed fragments per kilobase of transcript per million mapped reads (FPKM) values. The FPKM value of mRNA and lncRNA were transformed by log_2_ (FPKM+1). lncRNAs, long noncoding RNA. This information was used to draw heatmaps with hierarchical clustering. Heatmaps for hierarchical clustering (C: mRNA, D: lncRNA).

We performed principal component analysis (PCA) based on mRNAs or lncRNAs from our catalog. The results showed that the samples could be separated by developmental stage and grouped into two classes: i) samples from the suckling period (D0, D7, and D28) and ii) samples from the late development stages (D180 and 2Y) (Figure 3A). The PCA based on lncRNA expression replicated all of the aforementioned features (Figure 3B), suggesting that lncRNA expression patterns were very similar overall to those of mRNA. The different expression patterns could also be measured by hierarchical clustering analysis (Figure 3C, D). These results showed that the gene expression levels were highly correlated between biological replicates and confirmed that our experiment had good reliability. Overall, we produced a comprehensive catalog of lncRNAs and mRNAs in the peripheral blood of pigs, which can serve as a foundation for understanding coding and noncoding RNA functions in porcine peripheral blood during development.

### Temporal gene expression patterns of mRNA and lncRNA

**Figure 4:**
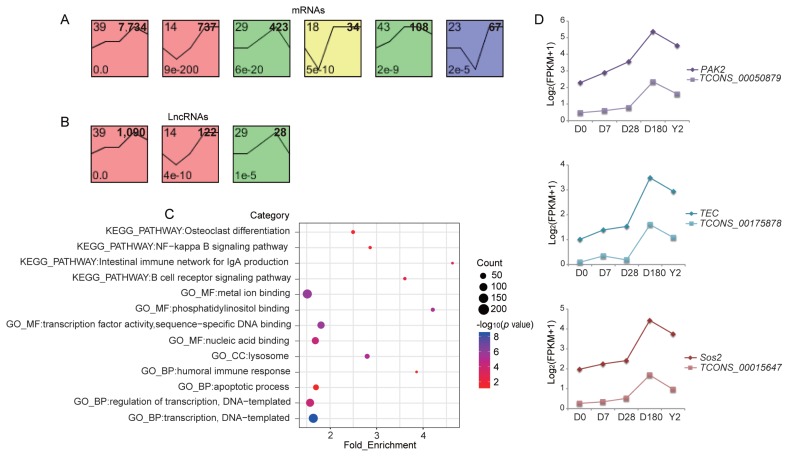
Expression patterns of the time-series modules and the lncRNA-mRNA pairs. STEM identified the temporal expression profiles of (A) mRNAs and (B) lncRNAs with p<0.05. The profile number in the top left corner of each profile box was assigned by STEM, the number in the bottom left represents the adjusted p value, and the number in the top right corner represents the cardinality of each cluster. (C) Functional categories (Benjamini-corrected p value<0.05) of mRNAs with the value of the fold change in the top 20% between D0 to D28 and D180 to Y2. (D) Dynamic expression profiles of mRNAs associated with the T-cell-receptor signaling pathway and the corresponding lncRNAs. lncRNAs, long noncoding RNA. D0, just after birth; D7, 7 days old; D28, 28 days old; D180, 180 days old; Y2, 2 years old.

To illustrate the dynamic gene expression pattern during blood development, we normalized the sequencing data to D0 and determined the temporal expression profiling of lncRNAs and mRNAs using STEM. Six temporal mRNA profiles were statistically significant within the 50 model profiles (Figure 4A). Genes in profiles 39, 29, and 43 were gradually increased from D0 to D180, but slightly decreased after D180. Genes in profiles 14, 18, and 23 showed expression patterns involving an initial drop, then a rise. Notably, profile 39 was the largest, containing approximately 60% of all mRNAs. Combining gene number and significance level, we selected genes from profile 39 to perform functional enrichment analyses. The results showed that these genes were associated with cellular components, protein processing, lysosomes, and metabolic pathways. Additionally, genes in profile 39 were expressed at low levels during D0 to D28 stage, but at higher level during D180 to Y2. Therefore, we determined the functions of the genes with the greatest expression level variations between the two developmental periods. For this, we calculated the fold change in expression levels of 7,743 mRNAs between the two stages and used mRNAs with a fold change in the top 20% to perform functional enrichment analysis. The significantly enriched functional categories for this set were mainly associated with regulating transcription, immune function, and apoptotic processes (Figure 4C). These may be the major biological processes that were altered during the two developmental stages in peripheral blood. Among genes related to humoral immune response, membrane spanning 4-domains A1 (also termed *CD20*) (log_2_ [fold change] = 3.4, p = 1.11E^−04^) was highly expressed at 180 days and 2 years and was one of the first B-cell differentiation antigens identified. *CD20* deficiency has been reported to cause a novel type of humoral immunodeficiency [17]. Among genes related to the B-cell-receptor signaling pathway, B cell linker (*BLNK*) (log_2_[fold change] = 2.6, p = 5.19E^−06^), was highly expressed at 180 days and 2 years. Previous studies showed that the BLNK adaptor protein has a direct and nonredundant role in pre-BII-cell differentiation and immunoglobulin (Ig) light chain gene rearrangements in humans [18].

Additionally, we found three statistically significant lncRNA temporal profiles were within the 50 model profiles (Figure 4B). Expression of lncRNAs in clusters 39 and 29 gradually increased from D0 to D180 and slightly decreased after D180. lncRNAs in cluster 14 showed downregulated expression from D0 to D7 and upregulated expression from D7 to 2Y. Surprisingly, most lncRNAs and mRNAs showed the same temporal expression patterns, implying that their expression patterns were highly correlated during development. This may indicate functional relatedness or a regulatory relationship between them. To test this hypothesis, we calculated Pearson correlations between 7,743 mRNAs and 1,090 lncRNAs in profile 39, and approximately 63% of the lncRNA-mRNA pairs were highly correlated (|r|>0.95, p<0.05). Among these highly correlated pairs, 614 were situated within 100 kb of each other, among which, 199 pairs actually overlapped (Supplementary Table S2). We identified three mRNAs associated with the T-cell-receptor signaling pathway, and the corresponding lncRNA-mRNA pairs were *TCONS_00050879-PAK2* (|r| = 0.97, p = 2.47E^−06^), *TCONS_00175878-TEC* (|r| = 0.98, p = 1.40E^−06^), and *TCONS_00015647-Sos2* (|r| = 0.97, p = 2.14E^−06^) (Figure 4D). Some researchers demonstrated that in primary T-cells, dominant-negative p21-activated kinase (*PAK2*) prevented anti-CD3/CD28-induced interleukin 2 (IL-2) production and T cell antigen receptor (TCR)-induced CD40 ligand expression, both of which are key functions of activated T-cells [19]. Tec protein tyrosine kinase (TEC) mediated signals negatively regulate *CD25* expression induced by TCR cross-linking, implying that this protein tyrosine kinase (*PTK*) plays a role in attenuating of IL-2 activity in human T lymphocytes [20]. Baltanas et al [21] demonstrated functional redundancy between stroke outcomes study 1 (*Sos1*) and stroke outcomes study 2 (*Sos2*) for organismal homeostasis and survival and for T and B lymphocytes development and maturation. These lncRNAs may regulate the expression of corresponding mRNAs or share regulatory regions within their gene loci that coordinate their expression.

### Functional enrichment analysis of differentially expressed mRNA and lncRNA

**Figure 5:**
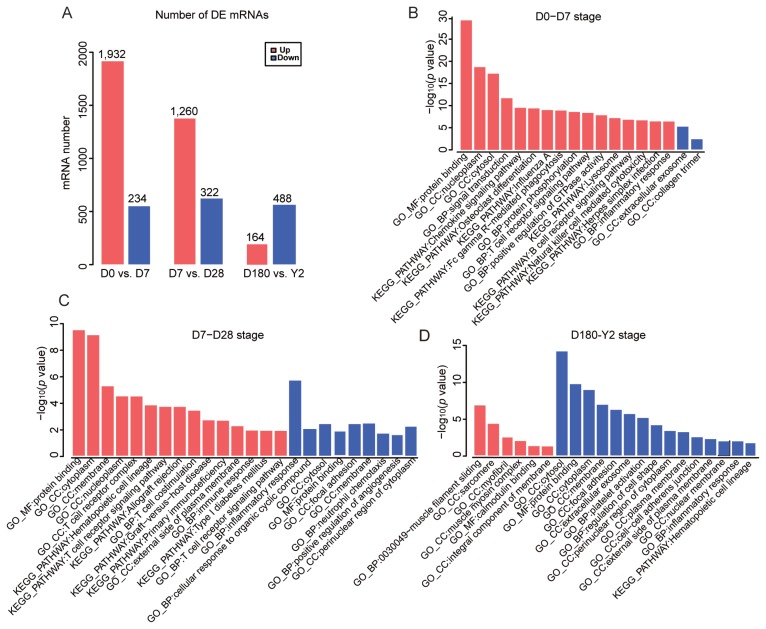
The number and functional enrichment analysis of the differentially expressed (DE) mRNAs (A) Numbers of DE upregulated (red) and DE downregulated (blue) mRNAs in porcine peripheral blood between adjacent time points in the suckling period (D0 vs D7, and D7 vs D28) and the late phase of development (D180 vs Y2). The main enriched categories (Benjamini-corrected p value<0.05 for DE upregulated (red) and downregulated (blue) mRNAs at (B) D0 to D7, (C) D7 to D28, and (D) D180 to 2Y. D0, just after birth; D7, 7 days old; D28, 28 days old; D180, 180 days old; Y2, 2 years old.

**Figure 6:**
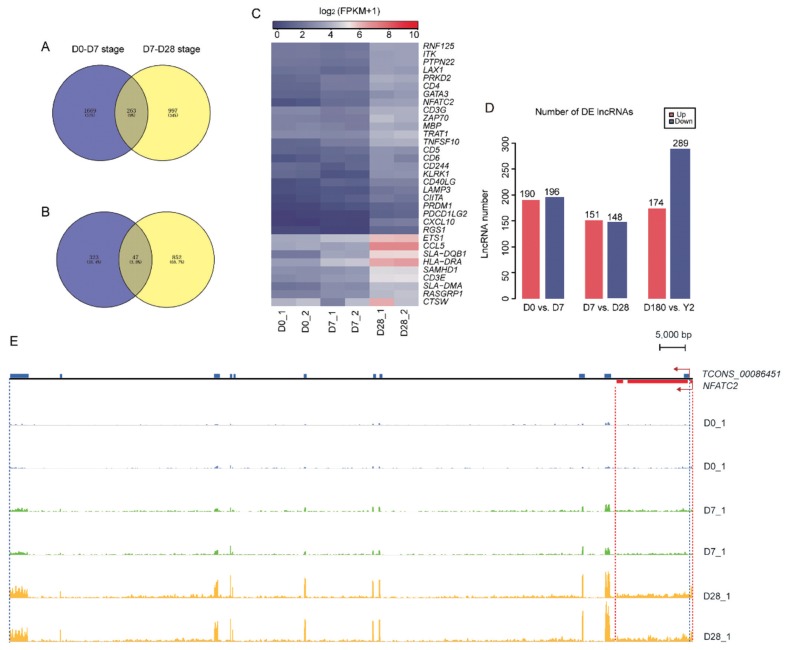
The number of DE lncRNAs and the loci and expression abundance of *TCONS_00086451* and *NFATC2*. Venn diagram of simultaneously (A) upregulated and (B) downregulated mRNAs at D0 to D7 and D7 to D28 stages. (C) Heatmap of continuously upregulated immune-related DE genes at D0 to D28. (D) Numbers of DE upregulated (red) and DE downregulated (blue) mRNAs between adjacent time points in the suckling period (D0 vs D7, and D7 vs D28) and the late phase of development (D180 vs Y2). (E) Loci and expression levels of *TCONS_00086451* and *NFATC2* read abundance which was generated by integrative genomics viewer (IGV) v.2.4.10 [30]. The blue rectangles represent exons of *TCONS_00086451* and the red rectangles represent exons of *NFATC2*. *NFATC2*, nuclear factor of activated T-cells cytoplasmic 2. D0, just after birth; D7, 7 days old; D28, 28 days old; D180, 180 days old; Y2, 2 years old.

As shown in the hierarchical clustering and STEM analysis, the five time points were grouped into two stages (the suckling period and the late phase of development). To better understand the changes in biological processes between adjacent time points within each stage, we first identified the DE genes (|log_2_ [fold change]|≥1.5, p<0.05) between adjacent time points (D0 vs D7 and D7 vs D28) in the suckling period (Figure 5A). At D0 to D7, 1,932 and 234 DE mRNAs were upregulated and downregulated, respectively. These upregulated genes were mainly enriched in categories associated with protein binding, cellular components, signal transduction, and immune response. The downregulated mRNAs were only enriched in two processes related to extracellular exosomes and collagen trimers (Figure 5B). At D7 to D28, 1,260 DE mRNAs were upregulated, which were involved in processes related to protein binding, cellular components, immune response, and autoimmune and immunodeficiency diseases. In contrast, 322 DE mRNAs were downregulated, which were mainly enriched in categories related to inflammatory response and neutrophil chemotaxis (Figure 5C). Venn diagram analysis showed that 263 and 47 mRNAs were simultaneously upregulated and downregulated at D0 to D7 and D7 to D28 stages, respectively. (Figure 6A, B). Surprisingly, these continuously upregulated genes were enriched in many immune-related processes (Supplementary Table S3), especially those associated with T-cells, such as T-cell costimulation, adaptive immune response, the T-cell-receptor signaling pathway, the T-cell-receptor complex, and MHC class II protein complex and antigen processing. This suggests that the genes in these processes (e.g., RAS guanyl releasing protein 1, nuclear factor of activated T-cells cytoplasmic 2 [*NFATC2*], *CD5*, and *CD6*; Figure 6C) are crucial to developing a healthy blood-based immune system during lactation. As the number of simultaneously downregulated mRNAs was small, no significant categories were enriched for these mRNAs. In addition, we identified the DE mRNAs between adjacent time points in the late phase of development (D180 vs Y2; Figure 5A). One hundred sixty-four upregulated mRNAs were mainly involved in processes related to regulating muscle composition and movement, such as muscle filament sliding, sarcomeres, and myofibrils, and 488 downregulated mRNAs were related to protein binding, cellular components, platelet activation, and the hematopoietic cell lineage (Figure 5D).

Our research also identified 980 DE lncRNAs in the three comparison groups (D0 vs D7, D7 vs D28, and D180 vs Y2) (Figure 6D). Among them, the expression levels of 300 adjacently located mRNAs (100 kb up- and downstream) were highly correlated with 95 DE lncRNAs (|r|>0.95 and p<0.05). (Supplementary Table S4) We considered that these mRNAs may have similar structures or functions to the corresponding lncRNAs [14]. Notably, among these lncRNA-mRNA pairs, lncRNA *TCONS-00086451* was a sense-overlapping lncRNA of *NFATC2* (Supplementary Table S5). This lncRNA-mRNA pair was continuously upregulated in the peripheral blood from D0 to D28 (Figure 6E), with a Pearson correlation coefficient of 0.98 (p = 3.12E^−07^). NFATC2 is a transcription factor that plays a critical role in regulating lymphocyte effector differentiation. Moreover, *NFATC2* is an important factor controlling mast cell accessory function at the interface of innate and adaptive immunity [22]. Consequently, *TCONS-00086451* may be associated with *NFATC2* and appears to play a role in adaptive immune system development.

### Validation of differentially expressed genes

**Figure 7:**
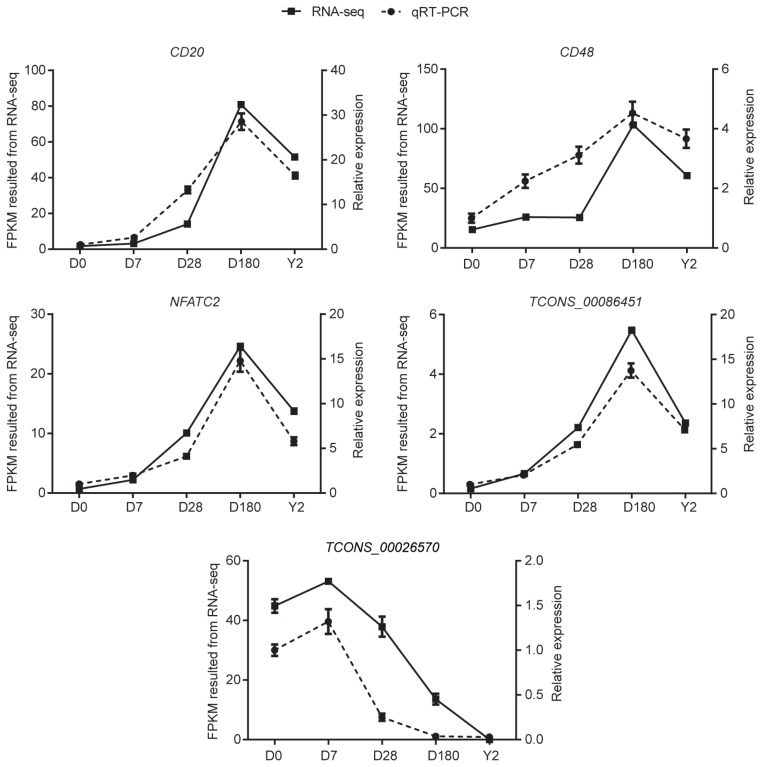
Verification of differentially expressed genes. Validation of three differentially expressed mRNAs (*CD20*, *CD48*, and *NFATC2*) and two differentially expressed lncRNAs (*TCONS_00026570* and *TCONS_00086451*) by qRT-PCR. Data are the mean±standard error of the mean. *NFATC2*, nuclear factor of activated T-cells cytoplasmic 2; qRT-PCR, quantitative real-time polymerase chain reaction.

We selected five candidate DE genes (including *CD20*, *NFATC2*, and *TCONS-00086451*), and verified their expression patterns from our RNA-seq results via qRT-PCR. The results confirmed that the five DE genes were expressed at all five development stages (Figure 7). In addition, qPCR confirmed that the expression patterns of the five DE genes were consistent with their expression levels calculated from the RNA-seq data. These results indicate that most of the identified lncRNAs and mRNAs were truly expressed *in vivo*.

## DISCUSSION

Health issues are a major and prevalent problem in the swine industry, causing serious economic and welfare-related problems. The immune system plays a crucial role in disease resistance among animals. The body’s immune status can be best monitored by measuring transcript abundances in the blood [5]. The blood development process should be clarified postnatally to better regulate the immune statuses of pigs and minimize illness-associated economic losses. However, compared with the substantial research on the transcriptional profiles in many porcine tissues, limited studies have focused on analyzing the postnatal blood transcriptome in pigs. Thus, in the present study, we produced a catalog of lncRNAs and mRNAs from porcine peripheral blood at different time points from the newborn stage to adult life using RNA-seq data.

Ou r PCA analysis results showed that the five time points (D0, D7, D28, D180, and Y2) were divided into two developmental stages: the suckling period (D0, D7, and D28) and the late development phase (D180 and Y2). Time points within the same stage had similar snapshots of gene expression. Similarly, using a STEM platform to investigate how gene expression profiles change continuously over time during blood development in pigs showed that most mRNAs in the blood were expressed at low level during the suckling period but at higher levels later in development. Thus, we suggest that genes expressed during each time point within a stage should have similar underlying regulatory mechanisms. We also found that both the number of expressed genes and the gene expression levels in the blood peaked at 180 days among the five time points, indicating that most genes may be activated at 180 days. This can be caused by two factors: i) regulation of gene transcriptional activity and ii) relative changes in the abundances of cell populations expressing transcripts at constant levels [5]. Time-series analysis also showed that mRNAs and lncRNAs had similar expression patterns, and most were highly correlated (|r|>0.95 and p< 0.05). Most evidence suggests that lncRNA expression can modulate and be highly correlated with neighboring mRNA expression in animal and plants [23,24]. We identified several lncRNA-mRNA pairs at overlapping loci that were highly correlated (|r|>0.95, p<0.05), such as *TCONS_00050879-PAK2*, *TCONS_00175878-TEC*, and *TCONS_00015647-Sos2*. These mRNAs were related to the T-cell-receptor signaling pathway, the lncRNAs may promote blood-based immune system development via co-regulation with these immune-related mRNAs.

Changes in the biological processes between adjacent time points during lactation revealed that the DE genes associated with inflammatory response and viral and bacterial infections were significantly upregulated in the peripheral blood after birth. This is consistent with previous reports describing that neonates have little immunological memory, which increases their vulnerability to infectious agents [25]. Notably, many immune-related genes, especially those associated with T-cells, were continuously upregulated during lactation, indicating that the adaptive immune system of the piglets was being established at this stage. These results are consistent with the findings of previous studies describing that the total percentages of γδT-cells showed significant age-related increases in the piglet blood during the first month of life [2]. For example, we found that T-cell markers (*CD4*) and toll-like receptors (*TLR3* and *TLR7*) displayed age-related changes. *CD4* was significantly upregulated at D0 to D28. *TLR3* and *TLR7* were significantly upregulated at D0 to D7. T lymphocytes and TLRs are associated with resistance to pathogenic challenges and play important roles in the mammalian immune system [26]. Age-related changes and distribution of T-cell markers and TLRs have also been reported in the duck lymphoid organs [27]. In contrast, blood immune-related function remains a relatively stable state at the late stage of development (D180 to Y2), as few DE genes were immune-related in this period. Overall, these findings suggest that the immune system matures age-dependently at the early stage of development. Therefore, sufficient levels of proteins or amino acids should be supplied in the feed to ensure highly efficient antibody production.

Recent reports have revealed that many immune-related lncRNAs are located near or overlap with immune-responsible protein-coding genes such as interleukin 1b-regions of bidirectional transcription 46 [28] and *lnc-IL7R* [29]. These lncRNAs control their adjacent protein-coding genes via *cis*- or *trans*-acting mechanisms. Here, our results also indicate that DE lncRNA might co-regulate the normal development of blood-based immunity with immune-responsible mRNAs as the lncRNA-mRNA pairs (*TCON_S00086451-NFATC2*) show. The mRNAs overlap of the corresponding lncRNAs. However, the detailed regulatory mechanisms of the mRNA and lncRNA interaction remains unclear and further research is needed to elucidate this relationship.

Here, we produced an extensive resource for lncRNAs and mRNAs expressed in porcine peripheral blood at five developmental stages and predicted that several lncRNAs and mRNAs have functional effects. Our results, from a combined analysis of mRNA functional enrichment and lncRNA functional prediction, suggest that lncRNAs might regulate blood-based immune system development by co-regulation with mRNAs.

## Figures and Tables

**Table 1:** Summary of data information

Sample name	Raw data (Gb)	Clean data (Gb)	Proportion of Q30 (%)	Raw reads	Clean reads	Clean ratio (%)	Map ratio (%)
D0_1	12.40	12.07	94.36	82,688,220	80,500,754	97.35	97.84
D0_2	13.46	12.97	93.22	89,797,096	86,494,186	96.32	97.49
D7_1	12.54	12.10	92.61	83,659,704	80,673,656	96.43	97.46
D7_2	12.63	12.29	94.16	84,205,842	81,965,386	97.34	96.85
D28_1	12.84	12.53	94.11	85,610,972	83,557,454	97.6	97.15
D28_2	13.52	12.98	92.94	90,134,910	86,577,838	96.05	96.71
D180_1	12.55	12.25	94.1	83,723,906	81,707,884	97.59	94.99
D180_2	12.98	12.64	92.75	86,540,692	84,273,212	97.38	94.56
Y2_1	12.59	12.06	94.74	83,984,532	80,461,134	95.8	95.98
Y2_2	13.12	12.73	94.57	87,485,840	84,869,688	97.01	95.91
