# Supplementary material for: Temporal expression profiling of long noncoding RNA and mRNA in the peripheral blood during porcine development
Source: Asian-Australas J Anim Sci. 2019 Aug 3;33(5):836–47. doi: 10.5713/ajas.19.0313 (PMC7206404; doi:10.5713/ajas.19.0313)
Supplement: Supplementary file 1 [file ajas-19-0313-suppl.pdf]

Table 1. Summary of data information

Sample Name	Raw Data (Gb)	Clean Data (Gb)	Proportion of Q30 (%)	Raw Reads	Clean Reads	Clean Ratio (%)	Map Ratio (%)
D0_1	12.40	12.07	94.36	82,688,220	80,500,754	97.35	97.84
D0_2	13.46	12.97	93.22	89,797,096	86,494,186	96.32	97.49
D7_1	12.54	12.10	92.61	83,659,704	80,673,656	96.43	97.46
D7_2	12.63	12.29	94.16	84,205,842	81,965,386	97.34	96.85
D28_1	12.84	12.53	94.11	85,610,972	83,557,454	97.6	97.15
D28_2	13.52	12.98	92.94	90,134,910	86,577,838	96.05	96.71
D180_1	12.55	12.25	94.1	83,723,906	81,707,884	97.59	94.99
D180_2	12.98	12.64	92.75	86,540,692	84,273,212	97.38	94.56
Y2_1	12.59	12.06	94.74	83,984,532	80,461,134	95.8	95.98
Y2_2	13.12	12.73	94.57	87,485,840	84,869,688	97.01	95.91

Supplemental Table 1. Primer sequences of the q-PCR experiments

Gene symbol	Primer sequences (5'-3')	Product length (bp)	Tm (℃)
TCONS_00026570	F: CTCCTCACAAGGCACTAAACC<br>R: TTGTCAGGCAAAGAGGGCTA	152	59.4
TCONS_00086451	F: GCTGTGAGTTTGCCTTTGGA<br>R: TGACCTGAAGTCGAACCCCT	215	59.4
NFATC2	F:GCGGTTCCCAAGACGAGTTC<br>R:GTCCAGTACATCGTCGGGTC	125	60
CD48	F: GAGTCTACAAATCGCCAATC<br>R: CACCGTCAGCATAATCTTCC	258	59.4
CD20	F: CTTCGATGGGCCCTACACAA<br>R: CTGCTGCCAGGAATGATCCA	204	59.4
β-*ACTIN**	F: CCTGCGGCATTCACGAAACTAC<br>R: ACAGCACCGTGTTGGCGTAGAG	87	60

* Represents internal control genes

Supplemental Table 2. LncRNA–mRNA pairs with highly related (|r|>0.95, *p*<0.05) expression levels and overlapped loci (0 kb).

LncRNA_transcript ID	Ensemble Gene ID	Gene symbol	LncRNA start	LncRNA end	mRNA start	mRNA end	Distance	Chromosome	r value	*p* value
TCONS_00087061	ENSSSCG00000020663	*KMT2C*	5006134	5009662	4993204	5277722	0	18	0.993	8.88E-09
TCONS_00088914	ENSSSCG00000023801	*LANCL2*	48552244	48554245	48550748	48593204	0	18	0.966	5.73E-06
TCONS_00088446	ENSSSCG00000038158	*THAP5*	36587032	36588126	36584386	36592905	0	18	0.95	2.55E-05
TCONS_00090064	ENSSSCG00000016549	*MKLN1*	17561988	17564976	17504858	17832503	0	18	0.991	3.13E-08
TCONS_00090763	ENSSSCG00000016661	*43715*	38129185	38130425	38082569	38183916	0	18	0.959	1.23E-05
TCONS_00087386	ENSSSCG00000013470	-	8890013	8890838	8883491	8896489	0	18	0.975	1.78E-06
TCONS_00087730	ENSSSCG00000016545	*CHCHD3*	16177740	16178473	16052623	16329393	0	18	0.969	3.75E-06
TCONS_00089333	ENSSSCG00000016409	*UBE3C*	1558253	1559564	1543777	1654356	0	18	0.989	5.87E-08
TCONS_00087453	ENSSSCG00000032266	*SLC37A3*	9421814	9424300	9383792	9442790	0	18	0.982	5E-07
TCONS_00083529	ENSSSCG00000025534	*CSE1L*	50750666	50750760	50744908	50797553	0	17	0.962	8.84E-06
TCONS_00085597	ENSSSCG00000007284	*NCOA6*	38201495	38202434	38114290	38221888	0	17	0.987	1.15E-07
TCONS_00083549	ENSSSCG00000007466	*SLC9A8*	51435334	51435842	51400910	51477217	0	17	0.974	1.82E-06
TCONS_00082617	ENSSSCG00000036081	*TBC1D20*	34764545	34765847	34760299	34779701	0	17	0.976	1.49E-06
TCONS_00082860	ENSSSCG00000007275	*RALY*	37446564	37479718	37447383	37542464	0	17	0.961	9.7E-06
TCONS_00084675	ENSSSCG00000031857	*SMIM19*	11538850	11550510	11549516	11569655	0	17	0.988	9E-08
TCONS_00086451	ENSSSCG00000007477	*NFATC2*	52874827	52890259	52743570	52907428	0	17	0.984	3.12E-07
TCONS_00081112	ENSSSCG00000006994	*PCM1*	5619132	5620960	5596385	5690349	0	17	0.972	2.5E-06
TCONS_00076357	ENSSSCG00000016911	*SKIV2L2*	34484525	34485355	34478227	34606096	0	16	0.952	2.21E-05
TCONS_00077079	ENSSSCG00000022478	*STK10*	52116279	52117548	52003736	52166161	0	16	0.965	6.52E-06
TCONS_00066931	ENSSSCG00000033367	*PLEKHA2*	47696991	47697166	47626108	47713346	0	15	0.955	1.71E-05

TCONS_00068943	ENSSSCG00000026940	*CASP10*	1.05E+08	1.05E+08	1.05E+08	1.05E+08	0	15	0.969	3.96E-06
TCONS_00073647	ENSSSCG00000016074	*ANKRD44*	1.01E+08	1.01E+08	1.01E+08	1.01E+08	0	15	0.991	3.24E-08
TCONS_00074903	ENSSSCG00000024373	*TRIP12*	1.31E+08	1.31E+08	1.31E+08	1.31E+08	0	15	0.95	2.54E-05
TCONS_00070039	ENSSSCG00000016291	*GIGYF2*	1.33E+08	1.33E+08	1.33E+08	1.33E+08	0	15	0.978	1.03E-06
TCONS_00071269	ENSSSCG00000015701	*MGAT5*	17892700	17892829	17544802	17919559	0	15	0.958	1.3E-05
TCONS_00071266	ENSSSCG00000015701	*MGAT5*	17580255	17581322	17544802	17919559	0	15	0.959	1.2E-05
TCONS_00068955	ENSSSCG00000028157	*CASP8*	1.05E+08	1.05E+08	1.05E+08	1.05E+08	0	15	0.985	2.43E-07
TCONS_00065924	ENSSSCG00000021343	*ZEB2*	7565885	7571320	7498897	7632655	0	15	0.991	3.09E-08
TCONS_00068615	ENSSSCG00000031954	*SLC39A10*	99659548	99666892	99584613	99724613	0	15	0.967	5E-06
TCONS_00058822	ENSSSCG00000037592	*SLF2*	1.12E+08	1.12E+08	1.12E+08	1.12E+08	0	14	0.983	3.85E-07
TCONS_00065269	ENSSSCG00000038222	*CACUL1*	1.29E+08	1.29E+08	1.29E+08	1.29E+08	0	14	0.969	4.12E-06
TCONS_00059186	ENSSSCG00000010628	*SHOC2*	1.21E+08	1.21E+08	1.21E+08	1.21E+08	0	14	0.976	1.4E-06
TCONS_00053997	ENSSSCG00000033113	*CHMP7*	7393360	7394060	7389791	7405751	0	14	0.964	7.15E-06
TCONS_00056886	ENSSSCG00000036476	*REEP3*	66967138	66969947	66966357	67071197	0	14	0.987	1.06E-07
TCONS_00064806	ENSSSCG00000010571	*C10orf76*	1.13E+08	1.13E+08	1.13E+08	1.13E+08	0	14	0.983	3.5E-07
TCONS_00052287	ENSSSCG00000011970	*CMSS1*	1.59E+08	1.59E+08	1.59E+08	1.59E+08	0	13	0.976	1.45E-06
TCONS_00040759	ENSSSCG00000011604	*CHCHD4*	70419847	70423881	70417326	70428922	0	13	0.992	2.1E-08
TCONS_00039356	ENSSSCG00000024513	*KLHL18*	30084626	30085471	30062836	30141511	0	13	0.978	9.28E-07
TCONS_00042939	ENSSSCG00000011775	*KLHL24*	1.22E+08	1.22E+08	1.22E+08	1.22E+08	0	13	0.986	1.76E-07
TCONS_00046752	ENSSSCG00000011340	*SMARCC1*	30491568	30492692	30407858	30576448	0	13	0.976	1.47E-06
TCONS_00040415	ENSSSCG00000011540	*SETD5*	65834980	65836517	65759965	65854432	0	13	0.966	5.32E-06
TCONS_00040595	ENSSSCG00000011575	*ATG7*	67431706	67431740	67417938	67699655	0	13	0.984	3.11E-07
TCONS_00049991	ENSSSCG00000011745	*PRKCI*	1.09E+08	1.09E+08	1.09E+08	1.09E+08	0	13	0.971	3.14E-06
TCONS_00050813	ENSSSCG00000011825	*ATP13A3*	1.31E+08	1.31E+08	1.31E+08	1.31E+08	0	13	0.979	8.36E-07
TCONS_00050879	ENSSSCG00000011843	*PAK2*	1.33E+08	1.33E+08	1.33E+08	1.33E+08	0	13	0.972	2.48E-06
TCONS_00032922	ENSSSCG00000034858	*RAP1GAP2*	48870740	48937581	48806715	49018115	0	12	0.997	1.95E-10

TCONS_00031158	ENSSSCG00000017406	*STAT5B*	20517658	20517949	20498096	20574353	0	12	0.985	2.45E-07
TCONS_00029856	ENSSSCG00000024235	*NPLOC4*	1254689	1254745	1251283	1288258	0	12	0.971	3.14E-06
TCONS_00030407	ENSSSCG00000037754	*SLC39A11*	8082410	8083143	7759613	8204762	0	12	0.971	3.14E-06
TCONS_00034365	ENSSSCG00000017169	*SEC14L1*	4486709	4488997	4441114	4486771	0	12	0.993	7.87E-09
TCONS_00033114	ENSSSCG00000017924	*PELP1*	52184453	52185952	52177243	52229370	0	12	0.966	5.6E-06
TCONS_00036045	ENSSSCG00000017592	*MBTD1*	27491727	27492163	27450806	27528894	0	12	0.958	1.24E-05
TCONS_00029992	ENSSSCG00000017167	*CYTH1*	3393448	3397169	3356224	3432644	0	12	0.987	1.1E-07
TCONS_00034900	ENSSSCG00000039909	*ICAM2*	14970910	14973198	14966077	14997437	0	12	0.985	2.06E-07
TCONS_00025538	ENSSSCG00000009384	*INTS6*	16393784	16396722	16372805	16468247	0	11	0.983	3.63E-07
TCONS_00026879	ENSSSCG00000009541	*ABHD13*	75553493	75556842	75547747	75570263	0	11	0.99	3.67E-08
TCONS_00026082	ENSSSCG00000009449	*TDRD3*	33276073	33279071	33258041	33440123	0	11	0.99	3.72E-08
TCONS_00027204	ENSSSCG00000026744	*ZDHHC20*	1344904	1345000	1327133	1405934	0	11	0.965	6.35E-06
TCONS_00025398	ENSSSCG00000037746	*NHLRC3*	14331451	14341248	14184134	14370915	0	11	0.987	1.33E-07
TCONS_00025398	ENSSSCG00000029855	*LHFPL6*	14331451	14341248	14319425	14576135	0	11	0.979	8.07E-07
TCONS_00025804	ENSSSCG00000035098	*MED4*	19553674	19556346	19551906	19569138	0	11	0.966	5.81E-06
TCONS_00025869	ENSSSCG00000009410	*RUBCNL*	20999887	21000287	20948641	21040802	0	11	0.97	3.35E-06
TCONS_00020128	ENSSSCG00000010987	*UBAP2*	32832654	32840086	32755978	32892869	0	10	0.98	6.32E-07
TCONS_00024193	ENSSSCG00000011133	*PFKFB3*	64850039	64859050	64776318	64859050	0	10	0.979	8.17E-07
TCONS_00024252	ENSSSCG00000011136	-	65258150	65259440	65196118	65267043	0	10	0.981	5.33E-07
TCONS_00022498	ENSSSCG00000010900	*DENND1B*	20542834	20550320	20392326	20707474	0	10	0.968	4.25E-06
TCONS_00020520	ENSSSCG00000011022	*SVIL*	41374940	41376374	41176358	41462764	0	10	0.983	3.45E-07
TCONS_00021033	ENSSSCG00000024127	*ABI1*	49008147	49012936	48939403	49069441	0	10	0.956	1.56E-05
TCONS_00019997	ENSSSCG00000010955	*GOLM1*	29160687	29161227	29117567	29219508	0	10	0.983	3.48E-07
TCONS_00020361	ENSSSCG00000028655	-	38989168	38989863	38970428	39004972	0	10	0.987	1.25E-07
TCONS_00021573	ENSSSCG00000011145	*ASB13*	65276790	65278614	65275467	65303072	0	10	0.956	1.62E-05
TCONS_00182550	ENSSSCG00000015345	*GLCCI1*	77936986	77992038	77915026	78334082	0	9	0.968	4.69E-06

TCONS_00184460	ENSSSCG00000015569	*SWT1*	1.26E+08	1.26E+08	1.26E+08	1.26E+08	0	9	0.987	1.21E-07
TCONS_00188637	ENSSSCG00000015336	*SLC25A13*	75787209	75789981	75763183	76306060	0	9	0.97	3.38E-06
TCONS_00182890	ENSSSCG00000038469	-	91966617	91968269	91901884	91971695	0	9	0.989	7.05E-08
TCONS_00184043	ENSSSCG00000015519	*RASAL2*	1.2E+08	1.2E+08	1.2E+08	1.2E+08	0	9	0.95	2.54E-05
TCONS_00189240	ENSSSCG00000015410	*PHTF2*	1.02E+08	1.02E+08	1.02E+08	1.02E+08	0	9	0.981	5.65E-07
TCONS_00189858	ENSSSCG00000040158	*SNORD75*	1.16E+08	1.16E+08	1.16E+08	1.16E+08	0	9	0.984	2.86E-07
TCONS_00188492	ENSSSCG00000015313	*KRIT1*	72168840	72169501	72144530	72185727	0	9	0.966	5.55E-06
TCONS_00187270	ENSSSCG00000015071	*SIK3*	44404795	44406214	44223232	44471717	0	9	0.962	9.06E-06
TCONS_00176845	ENSSSCG00000008990	*MRPL1*	73327636	73335755	73305439	73414563	0	8	0.972	2.51E-06
TCONS_00175901	ENSSSCG00000008826	*FRYL*	38544441	38545578	38397687	38697194	0	8	0.957	1.36E-05
TCONS_00175907	ENSSSCG00000008826	*FRYL*	38672110	38672263	38397687	38697194	0	8	0.963	8.07E-06
TCONS_00175904	ENSSSCG00000008826	*FRYL*	38639187	38639308	38397687	38697194	0	8	0.982	4.05E-07
TCONS_00175878	ENSSSCG00000008820	*TEC*	38165177	38169150	38051061	38181874	0	8	0.976	1.4E-06
TCONS_00171067	ENSSSCG00000036488	*KLF3*	30027028	30027255	30024830	30060982	0	8	0.969	3.89E-06
TCONS_00174358	ENSSSCG00000009226	*KLHL8*	1.32E+08	1.32E+08	1.32E+08	1.32E+08	0	8	0.956	1.53E-05
TCONS_00174665	ENSSSCG00000009256	*ANTXR2*	1.38E+08	1.38E+08	1.38E+08	1.38E+08	0	8	0.982	4.78E-07
TCONS_00169060	ENSSSCG00000002257	*MCTP2*	84928178	84928294	84725921	84962894	0	7	0.962	8.33E-06
TCONS_00166771	ENSSSCG00000001639	*TRERF1*	37438644	37439799	37333381	37554970	0	7	0.973	2.16E-06
TCONS_00166770	ENSSSCG00000001639	*TRERF1*	37356384	37361282	37333381	37554970	0	7	0.952	2.11E-05
TCONS_00159996	ENSSSCG00000001201	*ZKSCAN8*	21981437	21988237	21981150	22000834	0	7	0.989	6.34E-08
TCONS_00160780	ENSSSCG00000001556	*MAPK14*	31842195	31844891	31791398	31862031	0	7	0.952	2.12E-05
TCONS_00164566	ENSSSCG00000037558	*YY1*	1.21E+08	1.21E+08	1.21E+08	1.21E+08	0	7	0.993	1.14E-08
TCONS_00160722	ENSSSCG00000001538	*DEF6*	31095674	31196511	31161320	31216434	0	7	0.973	2.31E-06
TCONS_00160748	ENSSSCG00000001539	*PPARD*	31262405	31262451	31222487	31297939	0	7	0.97	3.24E-06
TCONS_00170080	ENSSSCG00000002461	*BTBD7*	1.15E+08	1.15E+08	1.15E+08	1.15E+08	0	7	0.993	1.28E-08
TCONS_00159266	ENSSSCG00000001061	*JARID2*	11358130	11425958	11303859	11602097	0	7	0.967	5.12E-06

TCONS_00162615	ENSSSCG00000026663	-	68110550	68112990	68106227	68116983	0	7	0.993	1.04E-08
TCONS_00166048	ENSSSCG00000001455	-	24910767	24911360	24868656	24914037	0	7	0.962	8.38E-06
TCONS_00160715	ENSSSCG00000001532	*UHRF1BP1*	30845448	30850825	30731361	30958640	0	7	0.98	6.77E-07
TCONS_00166993	ENSSSCG00000001710	*RUNX2*	40133231	40145369	40106513	40349398	0	7	0.995	2.85E-09
TCONS_00161168	ENSSSCG00000001641	*UBR2*	37661431	37664076	37655733	37784353	0	7	0.974	2.09E-06
TCONS_00159049	ENSSSCG00000033009	-	7349722	7350192	7328279	7422238	0	7	0.975	1.78E-06
TCONS_00161226	ENSSSCG00000001646	*BICRAL*	37879393	37891316	37853036	37933661	0	7	0.981	6.18E-07
TCONS_00170177	ENSSSCG00000002495	*SYNE3*	1.17E+08	1.17E+08	1.17E+08	1.17E+08	0	7	0.985	2.02E-07
TCONS_00160984	ENSSSCG00000039952	*ZFAND3*	33655484	33658558	33378286	33718030	0	7	0.963	8.12E-06
TCONS_00152917	ENSSSCG00000003104	*STRN4*	52624511	52627061	52601938	52627905	0	6	0.952	2.22E-05
TCONS_00155916	ENSSSCG00000037121	*KIAA0319L*	91718470	91719954	91605502	91747834	0	6	0.958	1.26E-05
TCONS_00148536	ENSSSCG00000003809	*JAK1*	1.47E+08	1.47E+08	1.47E+08	1.48E+08	0	6	0.979	8.25E-07
TCONS_00145811	ENSSSCG00000003398	*UBE4B*	70365206	70367128	70345801	70462430	0	6	0.979	8.59E-07
TCONS_00146860	ENSSSCG00000027686	*YTHDF2*	85717629	85721094	85716780	85751560	0	6	0.956	1.52E-05
TCONS_00145184	ENSSSCG00000036601	*ZNF805*	61617385	61617464	61610573	61677600	0	6	0.968	4.53E-06
TCONS_00149096	ENSSSCG00000037235	-	1.63E+08	1.63E+08	1.63E+08	1.63E+08	0	6	0.983	3.84E-07
TCONS_00154320	ENSSSCG00000003348	-	63739664	63741854	63727680	63755198	0	6	0.996	1.29E-09
TCONS_00147478	ENSSSCG00000021738	-	96841275	96841974	96770225	96847128	0	6	0.967	5.08E-06
TCONS_00147438	ENSSSCG00000003670	*RLF*	95886915	95908376	95900086	95994043	0	6	0.988	7.73E-08
TCONS_00148591	ENSSSCG00000038861	*TM2D1*	1.51E+08	1.51E+08	1.51E+08	1.51E+08	0	6	0.974	2.07E-06
TCONS_00149045	ENSSSCG00000003872	*EPS15*	1.61E+08	1.61E+08	1.61E+08	1.61E+08	0	6	0.992	1.83E-08
TCONS_00147533	ENSSSCG00000003679	-	98518047	98518431	98462827	98539647	0	6	0.985	2.07E-07
TCONS_00145824	ENSSSCG00000003398	*UBE4B*	70437909	70442259	70345801	70462430	0	6	0.988	8.38E-08
TCONS_00150455	ENSSSCG00000038843	*ST3GAL2*	13423517	13456455	13402345	13456455	0	6	0.965	6.14E-06
TCONS_00154759	ENSSSCG00000024794	*FBXO42*	75383707	75385258	75364444	75446099	0	6	0.953	1.98E-05
TCONS_00149097	ENSSSCG00000037235	-	1.63E+08	1.63E+08	1.63E+08	1.63E+08	0	6	0.987	1.08E-07

TCONS_00156255	ENSSSCG00000037264	*RAB31*	98385657	98385862	98278648	98385862	0	6	0.957	1.45E-05
TCONS_00149335	ENSSSCG00000039770	*SLC6A9*	1.67E+08	1.67E+08	1.67E+08	1.67E+08	0	6	0.986	1.79E-07
TCONS_00151524	ENSSSCG00000040286	*CYLD*	34090082	34091152	34058095	34121361	0	6	0.953	2.03E-05
TCONS_00143915	ENSSSCG00000003072	*ZNF283*	50655233	50655679	50650505	50678027	0	6	0.965	6.39E-06
TCONS_00134783	ENSSSCG00000000784	*LRRK2*	71818714	71820102	71800337	71944105	0	5	0.953	2.05E-05
TCONS_00138006	ENSSSCG00000026498	*R3HDM2*	22686766	22687262	22554399	22714591	0	5	0.968	4.56E-06
TCONS_00140191	ENSSSCG00000000816	*SENP1*	78453565	78453631	78423137	78476313	0	5	0.989	6.66E-08
TCONS_00138219	ENSSSCG00000031580	-	30482938	30483896	30474381	30485066	0	5	0.959	1.21E-05
TCONS_00133211	ENSSSCG00000000493	*FRS2*	33750203	33753015	33724546	33852952	0	5	0.983	3.68E-07
TCONS_00138491	ENSSSCG00000000502	*CNOT2*	34393860	34426670	34426399	34550088	0	5	0.965	6.62E-06
TCONS_00131155	ENSSSCG00000000036	*PACSIN2*	5932557	5934842	5823811	5985173	0	5	0.986	1.81E-07
TCONS_00132117	ENSSSCG00000029196	*DIP2B*	16494585	16496618	16481138	16601045	0	5	0.955	1.65E-05
TCONS_00134721	ENSSSCG00000000765	*IL17RA*	69462133	69464403	69461529	69480112	0	5	0.981	5.19E-07
TCONS_00125850	ENSSSCG00000005950	*PHF20L1*	8436279	8437425	8394953	8468265	0	4	0.974	1.88E-06
TCONS_00120395	ENSSSCG00000005943	*ST3GAL1*	7814572	7878390	7814273	7911047	0	4	0.957	1.42E-05
TCONS_00122021	ENSSSCG00000006218	*PDE7A*	68843625	68846480	68779422	68896583	0	4	0.95	2.53E-05
TCONS_00129780	ENSSSCG00000006785	*RAP1A*	1.09E+08	1.09E+08	1.09E+08	1.09E+08	0	4	0.981	6.17E-07
TCONS_00127577	ENSSSCG00000006206	-	68070779	68073232	67899254	68073232	0	4	0.955	1.73E-05
TCONS_00130256	ENSSSCG00000006870	-	1.18E+08	1.18E+08	1.18E+08	1.18E+08	0	4	0.972	2.66E-06
TCONS_00121093	ENSSSCG00000022479	*UBR5*	34661220	34666285	34645809	34794338	0	4	0.986	1.57E-07
TCONS_00120476	ENSSSCG00000028759	*ASAP1*	10342786	10347018	10103193	10451924	0	4	0.982	4.13E-07
TCONS_00126167	ENSSSCG00000024412	*RNF139*	15175081	15175636	15154332	15196922	0	4	0.989	7.42E-08
TCONS_00122137	ENSSSCG00000006233	*CA8*	73130721	73133279	73047875	73141990	0	4	0.983	4E-07
TCONS_00128485	ENSSSCG00000006398	*SLAMF8*	90663337	90663681	90662391	90673340	0	4	0.977	1.16E-06
TCONS_00121727	ENSSSCG00000006168	*PEX2*	59253094	59253910	59252337	59270116	0	4	0.977	1.26E-06
TCONS_00121726	ENSSSCG00000006168	*PEX2*	59256098	59265708	59252337	59270116	0	4	0.993	9.46E-09

TCONS_00122016	ENSSSCG00000006218	*PDE7A*	68846699	68848606	68779422	68896583	0	4	0.967	4.73E-06
TCONS_00115180	ENSSSCG00000027324	*C16orf62*	26111264	26116283	26038478	26157399	0	3	0.98	7.26E-07
TCONS_00113099	ENSSSCG00000008583	*ITSN2*	1.14E+08	1.14E+08	1.14E+08	1.14E+08	0	3	0.975	1.63E-06
TCONS_00107691	ENSSSCG00000007609	*ARPC1A*	6300536	6301128	6296241	6328672	0	3	0.972	2.69E-06
TCONS_00111218	ENSSSCG00000023409	*TIA1*	72252737	72254423	72240590	72277296	0	3	0.968	4.28E-06
TCONS_00119631	ENSSSCG00000008624	*LPIN1*	1.25E+08	1.25E+08	1.25E+08	1.25E+08	0	3	0.987	1.19E-07
TCONS_00113297	ENSSSCG00000008617	*FAM49A*	1.21E+08	1.21E+08	1.21E+08	1.21E+08	0	3	0.956	1.58E-05
TCONS_00113768	ENSSSCG00000034390	*CARD11*	2197531	2201399	2145180	2266355	0	3	0.979	7.94E-07
TCONS_00119307	ENSSSCG00000008581	*NCOA1*	1.14E+08	1.14E+08	1.14E+08	1.14E+08	0	3	0.983	3.23E-07
TCONS_00098530	ENSSSCG00000033213	*LYRM7*	1.34E+08	1.34E+08	1.33E+08	1.34E+08	0	2	0.953	2.09E-05
TCONS_00098532	ENSSSCG00000033213	*LYRM7*	1.34E+08	1.34E+08	1.33E+08	1.34E+08	0	2	0.996	1.52E-09
TCONS_00100457	ENSSSCG00000013052	-	8334255	8341306	8287802	8355129	0	2	0.977	1.24E-06
TCONS_00101284	ENSSSCG00000013298	*PDHX*	26046732	26046944	26015857	26094846	0	2	0.99	4.41E-08
TCONS_00093147	ENSSSCG00000013256	*ARHGAP1*	15847790	15849038	15834270	15857543	0	2	0.98	7.37E-07
TCONS_00095935	ENSSSCG00000013437	*TCF3*	76861307	76863247	76859074	76898350	0	2	0.952	2.19E-05
TCONS_00106899	ENSSSCG00000014387	-	1.43E+08	1.43E+08	1.43E+08	1.43E+08	0	2	0.986	1.79E-07
TCONS_00104428	ENSSSCG00000014033	*RMND5B*	80244586	80245947	80224871	80247065	0	2	0.983	3.91E-07
TCONS_00101105	ENSSSCG00000013284	*TTC17*	18892855	18919640	18807530	18954667	0	2	0.994	5.44E-09
TCONS_00098713	ENSSSCG00000014304	*SEC24A*	1.37E+08	1.37E+08	1.37E+08	1.37E+08	0	2	0.989	7.43E-08
TCONS_00098713	ENSSSCG00000014305	*SAR1B*	1.37E+08	1.37E+08	1.37E+08	1.37E+08	0	2	0.993	1.34E-08
TCONS_00106865	ENSSSCG00000028168	*TAF7*	1.43E+08	1.43E+08	1.43E+08	1.43E+08	0	2	0.953	2.01E-05
TCONS_00095866	ENSSSCG00000013457	*DOT1L*	76358306	76362312	76339442	76403354	0	2	0.969	4.11E-06
TCONS_00096624	ENSSSCG00000014088	*IQGAP2*	85366550	85370096	85293937	85633641	0	2	0.98	6.65E-07
TCONS_00105221	ENSSSCG00000024193	*LYSMD3*	97696036	97697155	97689376	97703742	0	2	0.951	2.36E-05
TCONS_00101612	ENSSSCG00000013362	*UEVLD*	40615385	40617567	40617172	40673369	0	2	0.977	1.14E-06
TCONS_00105281	ENSSSCG00000027608	*FAM172A*	1.01E+08	1.01E+08	1E+08	1.01E+08	0	2	0.983	3.61E-07

TCONS_00099300	ENSSSCG00000014416	*TCERG1*	1.48E+08	1.48E+08	1.48E+08	1.48E+08	0	2	0.96	1.12E-05
TCONS_00018585	ENSSSCG00000038868	*GPR107*	2.7E+08	2.7E+08	2.7E+08	2.7E+08	0	1	0.985	2.06E-07
TCONS_00018586	ENSSSCG00000038868	*GPR107*	2.7E+08	2.7E+08	2.7E+08	2.7E+08	0	1	0.95	2.5E-05
TCONS_00002771	ENSSSCG00000004405	*FIG4*	75750654	75751000	75693531	75847515	0	1	0.962	8.33E-06
TCONS_00015466	ENSSSCG00000004989	*FBXO33*	1.7E+08	1.7E+08	1.7E+08	1.7E+08	0	1	0.973	2.35E-06
TCONS_00005019	ENSSSCG00000004821	-	1.39E+08	1.39E+08	1.39E+08	1.39E+08	0	1	0.966	5.83E-06
TCONS_00010483	ENSSSCG00000004123	*UTRN*	20809450	20810018	20511240	21043642	0	1	0.959	1.18E-05
TCONS_00006821	ENSSSCG00000037813	-	2.01E+08	2.01E+08	2.01E+08	2.01E+08	0	1	0.982	4.61E-07
TCONS_00005645	ENSSSCG00000004958	*PIAS1*	1.66E+08	1.66E+08	1.66E+08	1.66E+08	0	1	0.956	1.56E-05
TCONS_00003126	ENSSSCG00000004473	*PHIP*	87218923	87220433	87117224	87242674	0	1	0.984	2.59E-07
TCONS_00017257	ENSSSCG00000005343	*RNF38*	2.37E+08	2.37E+08	2.37E+08	2.37E+08	0	1	0.961	9.94E-06
TCONS_00003997	ENSSSCG00000004610	*CCPG1*	1.16E+08	1.16E+08	1.16E+08	1.17E+08	0	1	0.95	2.49E-05
TCONS_00014533	ENSSSCG00000004826	*SELENOS*	1.4E+08	1.4E+08	1.4E+08	1.4E+08	0	1	0.959	1.17E-05
TCONS_00004059	ENSSSCG00000004617	*FAM214A*	1.19E+08	1.19E+08	1.19E+08	1.19E+08	0	1	0.995	3.31E-09
TCONS_00006487	ENSSSCG00000005092	*SLC38A6*	1.9E+08	1.9E+08	1.9E+08	1.9E+08	0	1	0.993	9.87E-09
TCONS_00015647	ENSSSCG00000005015	*SOS2*	1.8E+08	1.8E+08	1.8E+08	1.8E+08	0	1	0.973	2.14E-06
TCONS_00015219	ENSSSCG00000004952	*SMAD3*	1.65E+08	1.65E+08	1.65E+08	1.65E+08	0	1	0.962	8.48E-06
TCONS_00193155	ENSSSCG00000037775	-	3673879	3674346	3591646	3841358	0	AEMK02000452.1	0.978	9.25E-07
TCONS_00193761	ENSSSCG00000038870	*TNRC6A*	695735	695776	580337	823839	0	AEMK02000510.1	0.951	2.32E-05
TCONS_00192752	ENSSSCG00000002525	*TRAF3*	1529117	1535475	1512021	1627281	0	AEMK02000452.1	0.972	2.57E-06

TCONS_00202439	ENSSSCG00000012434	*ATRX*	61737166	61738548	61583574	61872266	0	X	0.991	2.83E-08
TCONS_00202436	ENSSSCG00000012434	*ATRX*	61648628	61650115	61583574	61872266	0	X	0.952	2.28E-05
TCONS_00203977	ENSSSCG00000012713	*ATP11C*	1.15E+08	1.15E+08	1.14E+08	1.15E+08	0	X	0.986	1.46E-07

Supplemental Table 3. The immune-related DE mRNAs simultaneously up-regulated during D0-D28

Category	Term	Gene symbol	Benjamini-adjusted *p* value	FDR
GO_BP	T cell costimulation	*HLA-DRA*/*CD3G*/*SPN*/*PDCD1LG2*/*KLRK1*/*HLA-DRB5*/ *HLA-DQB2*/*HLA-DQA1*/*CD4*/*CD40LG*/*CD5*/*CD3E*	3.24547E-06	4.43719E-06
GO_BP	Immune response	*HLA-DRA*/*CD1A*/*RGS1*/*ZAP70*/*SwPN*/*HLA-DQB2*/*HLA-DRB5*/*HLA-DQA1*/*HLA-B*/*GBP2*/*MBP*/*TNFSF10*/*SAMHD1*/*HLA-DMA*/*ETS1*/*PDCD1LG2*/*CTSW*/*CCL5*/*CD4*/*CD40LG*/*CIITA*/*CXCL10*/*LAX1*	6.6621E-06	1.82168E-05
GO_BP	Adaptive immune response	*PRDM1*/*CD1A*/*TRAT1*/*ZAP70*/*CD244*/*RNF125*/*CD6/KLRK1*/*LAMP3*/*CD4*/*PRKD2*/*LAX1*/*ITK*	0.000112354	0.000460855
GO_BP	T cell receptor signaling pathway	*HLA-DRA*/*ZAP70*/*TRAT1*/*CD3G*/*HLA-DQB2*/*HLA-DRB5*/*HLA-DQA1*/*PTPN22*/*PRKD2*/*ITK/CD3E*/*GATA3*/*CD4*	0.000112354	0.000460855
GO_CC	T cell receptor complex	*TRAT1*/*ZAP70*/*CD3G*/*CD6*/*CD4*/*CD3E*	0.000379578	0.002034654
GO_CC	MHC class II protein complex	*HLA-DRA*/*HLA-DMA*/*HLA-DRB5*/*HLA-DQB2*/ *HLA-DQA1*	0.006755444	0.145204633
GO_BP	Positive regulation of T cell proliferation	*SPN*/*PDCD1LG2*/*CD6*/*CCL5*/*CD4*/*CD40LG*/*CD3E*	0.017815868	0.147355148
KEGG_	Antigen processing and presentation	*HLA-DRA*/*HLA-DMA*/*HLA-DRB5*/*HLA-DQA1*/*CD4*/	0.024233634	1.470196272

PATHWAY		*CIITA*/*HLA-B*		
KEGG_ PATHWAY	T cell receptor signaling pathway	*ZAP70*/*CD3G*/*RASGRP1*/*NFATC2*/*CD4*/*CD40LG*/*ITK*/ *CD3E*	0.02597025	1.184423026

Supplemental Table 4. Information of DE lncRNAs and their highly correlated mRNAs.

LncRNA transcript ID	Ensemble Gene ID	Gene symbol	LncRNA start	LncRNA end	mRNA start	mRNA end	Distance	Chromosome	r value	*p* value
TCONS_00003670	ENSSSCG00000004576	*RORA*	1.1E+08	1.1E+08	1E+08	1E+08	70311	1	0.99498	2.8E-09
TCONS_00003719	ENSSSCG00000004576	*RORA*	1.1E+08	1.1E+08	1E+08	1E+08	38997	1	0.96252	8.3E-06
TCONS_00003719	ENSSSCG00000032194	*uc_338*	1.1E+08	1.1E+08	1E+08	1E+08	46790	1	0.96066	0.00001
TCONS_00006821	ENSSSCG00000037813	-	2E+08	2E+08	2E+08	2E+08	0	1	0.98188	4.6E-07
TCONS_00008028	ENSSSCG00000005322	*NPR2*	2.4E+08	2.4E+08	2E+08	2E+08	17334	1	0.95596	1.6E-05
TCONS_00008028	ENSSSCG00000025842	*SPAG8*	2.4E+08	2.4E+08	2E+08	2E+08	34936	1	0.97695	1.2E-06
TCONS_00011565	ENSSSCG00000004293	*SNX14*	5.5E+07	5.5E+07	5E+07	5E+07	83053	1	0.97429	1.9E-06
TCONS_00018585	ENSSSCG00000038868	*GPR107*	2.7E+08	2.7E+08	3E+08	3E+08	0	1	0.9852	2.1E-07
TCONS_00018586	ENSSSCG00000038868	*GPR107*	2.7E+08	2.7E+08	3E+08	3E+08	0	1	0.95037	2.5E-05
TCONS_00020970	ENSSSCG00000026354	-	4.8E+07	4.8E+07	5E+07	5E+07	65916	10	0.96158	9.1E-06
TCONS_00021573	ENSSSCG00000011145	*ASB13*	6.5E+07	6.5E+07	7E+07	7E+07	0	10	0.95551	1.6E-05
TCONS_00021573	ENSSSCG00000011137	*GDI2*	6.5E+07	6.5E+07	7E+07	7E+07	80334	10	0.9533	2E-05
TCONS_00022498	ENSSSCG00000010900	*DENND1B*	2.1E+07	2.1E+07	2E+07	2E+07	0	10	0.9683	4.3E-06
TCONS_00024193	ENSSSCG00000011133	*PFKFB3*	6.5E+07	6.5E+07	6E+07	6E+07	0	10	0.97908	8.2E-07
TCONS_00024193	ENSSSCG00000022849	*IL2RA*	6.5E+07	6.5E+07	6E+07	7E+07	89467	10	0.96535	6.1E-06

TCONS_00025398	ENSSSCG00000037746	*NHLRC3*	1.4E+07	1.4E+07	1E+07	1E+07	0	11	0.98675	1.3E-07
TCONS_00025398	ENSSSCG00000029855	*LHFPL6*	1.4E+07	1.4E+07	1E+07	1E+07	0	11	0.97915	8.1E-07
TCONS_00025804	ENSSSCG00000035098	*MED4*	2E+07	2E+07	2E+07	2E+07	0	11	0.9657	5.8E-06
TCONS_00030345	ENSSSCG00000017233	*RAB37*	6528720	6530943	6E+06	6E+06	35309	12	0.95738	1.4E-05
TCONS_00031533	ENSSSCG00000017517	*TBX21*	2.4E+07	2.4E+07	2E+07	2E+07	0	12	0.97008	3.4E-06
TCONS_00031642	ENSSSCG00000017543	*CALCOCO2*	2.5E+07	2.5E+07	3E+07	3E+07	3274	12	0.9778	1E-06
TCONS_00034900	ENSSSCG00000039909	*ICAM2*	1.5E+07	1.5E+07	1E+07	1E+07	0	12	0.98521	2.1E-07
TCONS_00034900	ENSSSCG00000017282	*SCN4A*	1.5E+07	1.5E+07	2E+07	2E+07	28245	12	0.99245	1.4E-08
TCONS_00034900	ENSSSCG00000032233	-	1.5E+07	1.5E+07	2E+07	2E+07	71606	12	0.97818	9.7E-07
TCONS_00037045	ENSSSCG00000040732	*WDR81*	4.8E+07	4.8E+07	5E+07	5E+07	94828	12	0.98219	4.3E-07
TCONS_00037045	ENSSSCG00000040205	*TLCD2*	4.8E+07	4.8E+07	5E+07	5E+07	76806	12	0.96151	9.2E-06
TCONS_00037136	ENSSSCG00000017866	*TAX1BP3*	5E+07	5E+07	5E+07	5E+07	33881	12	0.95156	2.3E-05
TCONS_00037136	ENSSSCG00000017867	*EMC6*	5E+07	5E+07	5E+07	5E+07	32845	12	0.95935	1.1E-05
TCONS_00037136	ENSSSCG00000031821	*HASPIN*	5E+07	5E+07	5E+07	5E+07	25097	12	0.97441	1.8E-06
TCONS_00045710	ENSSSCG00000039085	-	2.1E+08	2.1E+08	2E+08	2E+08	67448	13	0.96033	1E-05
TCONS_00052287	ENSSSCG00000011970	*CMSS1*	1.6E+08	1.6E+08	2E+08	2E+08	0	13	0.97582	1.5E-06
TCONS_00052287	ENSSSCG00000011972	*FILIP1L*	1.6E+08	1.6E+08	2E+08	2E+08	0	13	0.97399	1.9E-06
TCONS_00054699	ENSSSCG00000009786	*HIP1R*	3E+07	3E+07	3E+07	3E+07	12747	14	0.97657	1.3E-06
TCONS_00056552	ENSSSCG00000010179	*ARV1*	5.9E+07	5.9E+07	6E+07	6E+07	17506	14	0.97696	1.2E-06
TCONS_00059175	ENSSSCG00000010628	*SHOC2*	1.2E+08	1.2E+08	1E+08	1E+08	27914	14	0.98702	1.2E-07
TCONS_00059175	ENSSSCG00000010626	*RBM20*	1.2E+08	1.2E+08	1E+08	1E+08	52827	14	0.98608	1.6E-07
TCONS_00061865	ENSSSCG00000010033	*PRR14L*	4.8E+07	4.8E+07	5E+07	5E+07	0	14	0.99668	5.3E-10
TCONS_00061865	ENSSSCG00000010031	-	4.8E+07	4.8E+07	5E+07	5E+07	90513	14	0.95424	1.8E-05
TCONS_00065924	ENSSSCG00000021343	*ZEB2*	7565885	7571320	7E+06	8E+06	0	15	0.99081	3.1E-08
TCONS_00071266	ENSSSCG00000015701	*MGAT5*	1.8E+07	1.8E+07	2E+07	2E+07	0	15	0.9588	1.2E-05
TCONS_00079233	ENSSSCG00000016918	*MAP3K1*	3.6E+07	3.6E+07	4E+07	4E+07	76172	16	0.96382	7.2E-06

TCONS_00081131	ENSSSCG00000006995	*ASAH1*	5764279	5765424	6E+06	6E+06	13495	17	0.95775	1.3E-05
TCONS_00082324	ENSSSCG00000007135	-	3.1E+07	3.1E+07	3E+07	3E+07	24495	17	0.96807	4.4E-06
TCONS_00083139	ENSSSCG00000007337	*CTNNBL1*	4.1E+07	4.1E+07	4E+07	4E+07	0	17	0.96019	1.1E-05
TCONS_00083549	ENSSSCG00000007466	*SLC9A8*	5.1E+07	5.1E+07	5E+07	5E+07	0	17	0.97441	1.8E-06
TCONS_00084762	ENSSSCG00000028479	-	1.4E+07	1.4E+07	1E+07	1E+07	1352	17	0.96792	4.5E-06
TCONS_00086451	ENSSSCG00000007478	*ATP9A*	5.3E+07	5.3E+07	5E+07	5E+07	45910	17	0.98602	1.6E-07
TCONS_00086451	ENSSSCG00000007477	*NFATC2*	5.3E+07	5.3E+07	5E+07	5E+07	0	17	0.98358	3.1E-07
TCONS_00087297	ENSSSCG00000036013	-	7655995	7657112	8E+06	8E+06	0	18	0.96527	6.1E-06
TCONS_00087453	ENSSSCG00000032266	*SLC37A3*	9421814	9424300	9E+06	9E+06	0	18	0.98151	5E-07
TCONS_00088357	ENSSSCG00000016652	*LRRN3*	3.4E+07	3.4E+07	3E+07	3E+07	43452	18	0.96786	4.5E-06
TCONS_00088898	ENSSSCG00000016715	*OSBPL3*	4.7E+07	4.7E+07	5E+07	5E+07	0	18	0.9585	1.2E-05
TCONS_00092391	ENSSSCG00000033757	*FKBP2*	7787638	7796421	8E+06	8E+06	68822	2	0.99708	3.2E-10
TCONS_00096006	ENSSSCG00000027565	*ARHGAP45*	7.7E+07	7.7E+07	8E+07	8E+07	58540	2	0.97854	9.1E-07
TCONS_00096006	ENSSSCG00000021472	*POLR2E*	7.7E+07	7.7E+07	8E+07	8E+07	47135	2	0.96064	0.00001
TCONS_00096006	ENSSSCG00000023121	*ABCA7*	7.7E+07	7.7E+07	8E+07	8E+07	73597	2	0.95866	1.2E-05
TCONS_00096006	ENSSSCG00000013429	*STK11*	7.7E+07	7.7E+07	8E+07	8E+07	25048	2	0.99245	1.4E-08
TCONS_00096006	ENSSSCG00000039542	-	7.7E+07	7.7E+07	8E+07	8E+07	73599	2	0.95194	2.2E-05
TCONS_00098527	ENSSSCG00000033213	*LYRM7*	1.3E+08	1.3E+08	1E+08	1E+08	0	2	0.96641	5.4E-06
TCONS_00100457	ENSSSCG00000013052	-	8334255	8341306	8E+06	8E+06	0	2	0.97679	1.2E-06
TCONS_00103176	ENSSSCG00000027434	*OLFM2*	6.9E+07	6.9E+07	7E+07	7E+07	22230	2	0.98634	1.5E-07
TCONS_00103176	ENSSSCG00000033816	-	6.9E+07	6.9E+07	7E+07	7E+07	32775	2	0.96245	8.3E-06
TCONS_00109385	ENSSSCG00000007955	*CLUAP1*	3.9E+07	3.9E+07	4E+07	4E+07	24280	3	0.98422	2.7E-07
TCONS_00109385	ENSSSCG00000007956	*NLRC3*	3.9E+07	3.9E+07	4E+07	4E+07	1783	3	0.98733	1.1E-07
TCONS_00109515	ENSSSCG00000008039	*NTHL1*	4E+07	4E+07	4E+07	4E+07	87497	3	0.95276	2.1E-05
TCONS_00110822	ENSSSCG00000008232	*RNF181*	5.9E+07	5.9E+07	6E+07	6E+07	73760	3	0.95178	2.2E-05
TCONS_00113954	ENSSSCG00000033450	*PDAP1*	6296129	6296227	6E+06	6E+06	47597	3	0.95417	1.8E-05

TCONS_00113954	ENSSSCG00000007609	*ARPC1A*	6296129	6296227	6E+06	6E+06	14	3	0.9561	1.5E-05
TCONS_00113954	ENSSSCG00000034019	-	6296129	6296227	6E+06	6E+06	88533	3	0.99719	2.7E-10
TCONS_00113954	ENSSSCG00000007625	*ARPC1B*	6296129	6296227	6E+06	6E+06	33579	3	0.96748	4.7E-06
TCONS_00114945	ENSSSCG00000007811	*LAT*	1.9E+07	1.9E+07	2E+07	2E+07	43569	3	0.99417	5E-09
TCONS_00114945	ENSSSCG00000007812	*XPO6*	1.9E+07	1.9E+07	2E+07	2E+07	77887	3	0.9867	1.4E-07
TCONS_00117942	ENSSSCG00000008376	*COMMD1*	8E+07	8E+07	8E+07	8E+07	68139	3	0.97374	2E-06
TCONS_00117942	ENSSSCG00000008374	*B3GNT2*	8E+07	8E+07	8E+07	8E+07	10195	3	0.96553	5.9E-06
TCONS_00118905	ENSSSCG00000035747	*YIPF4*	1.1E+08	1.1E+08	1E+08	1E+08	7697	3	0.95271	2.1E-05
TCONS_00121727	ENSSSCG00000006168	*PEX2*	5.9E+07	5.9E+07	6E+07	6E+07	0	4	0.97665	1.3E-06
TCONS_00122137	ENSSSCG00000006233	*CA8*	7.3E+07	7.3E+07	7E+07	7E+07	0	4	0.98252	4E-07
TCONS_00124992	ENSSSCG00000006903	*RPAP2*	1.2E+08	1.2E+08	1E+08	1E+08	48907	4	0.97719	1.2E-06
TCONS_00125118	ENSSSCG00000006917	-	1.3E+08	1.3E+08	1E+08	1E+08	72579	4	0.95599	1.6E-05
TCONS_00126015	ENSSSCG00000039597	*PVT1_5*	1.2E+07	1.2E+07	1E+07	1E+07	20198	4	0.96375	7.2E-06
TCONS_00126055	ENSSSCG00000038194	*PVT1_3*	1.2E+07	1.2E+07	1E+07	1E+07	52638	4	0.98959	5.1E-08
TCONS_00126055	ENSSSCG00000038252	*PVT1_1*	1.2E+07	1.2E+07	1E+07	1E+07	8669	4	0.97223	2.5E-06
TCONS_00132590	ENSSSCG00000000382	*SMARCC2*	2.2E+07	2.2E+07	2E+07	2E+07	69081	5	0.96474	6.5E-06
TCONS_00132590	ENSSSCG00000000384	*NABP2*	2.2E+07	2.2E+07	2E+07	2E+07	70880	5	0.95806	1.3E-05
TCONS_00132590	ENSSSCG00000025190	*COQ10A*	2.2E+07	2.2E+07	2E+07	2E+07	35310	5	0.96263	8.2E-06
TCONS_00136275	ENSSSCG00000037910	*DUSP6*	9.3E+07	9.3E+07	9E+07	9E+07	92275	5	0.96026	1E-05
TCONS_00136457	ENSSSCG00000035733	*PLXNB2*	359131	361011	390363	418324	29352	5	0.98848	7.6E-08
TCONS_00136457	ENSSSCG00000000968	*SBF1*	359131	361011	260722	287455	71676	5	0.95212	2.2E-05
TCONS_00139387	ENSSSCG00000000650	*CLEC1B*	6.2E+07	6.2E+07	6E+07	6E+07	75734	5	0.96673	5.2E-06
TCONS_00143527	ENSSSCG00000002950	*SIPA1L3*	4.7E+07	4.7E+07	5E+07	5E+07	408	6	0.98988	4.5E-08
TCONS_00144203	ENSSSCG00000003119	*ZNF541*	5.3E+07	5.3E+07	5E+07	5E+07	80453	6	0.95724	1.4E-05
TCONS_00144203	ENSSSCG00000020791	*SNORD23*	5.3E+07	5.3E+07	5E+07	5E+07	62484	6	0.96637	5.4E-06
TCONS_00144203	ENSSSCG00000023371	*EHD2*	5.3E+07	5.3E+07	5E+07	5E+07	35147	6	0.95283	2.1E-05

TCONS_00144203	ENSSSCG00000034313	*SELENOW*	5.3E+07	5.3E+07	5E+07	5E+07	83984	6	0.98018	6.6E-07
TCONS_00149824	ENSSSCG00000032996	*SLC7A5*	1490054	1490319	2E+06	2E+06	55622	6	0.95675	1.5E-05
TCONS_00153181	ENSSSCG00000003193	*TBC1D17*	5.5E+07	5.5E+07	5E+07	5E+07	68294	6	0.98542	1.9E-07
TCONS_00153181	ENSSSCG00000003195	*PNKP*	5.5E+07	5.5E+07	5E+07	5E+07	94092	6	0.97966	7.3E-07
TCONS_00153181	ENSSSCG00000003201	*ATF5*	5.5E+07	5.5E+07	5E+07	5E+07	23355	6	0.98114	5.4E-07
TCONS_00153181	ENSSSCG00000003204	*VRK3*	5.5E+07	5.5E+07	5E+07	6E+07	0	6	0.97824	9.6E-07
TCONS_00153456	ENSSSCG00000039465	-	5.6E+07	5.6E+07	6E+07	6E+07	70303	6	0.9707	3.1E-06
TCONS_00154320	ENSSSCG00000003353	*SLC35E2B*	6.4E+07	6.4E+07	6E+07	6E+07	78217	6	0.97411	1.9E-06
TCONS_00154320	ENSSSCG00000003350	*MIB2*	6.4E+07	6.4E+07	6E+07	6E+07	47802	6	0.99493	2.9E-09
TCONS_00154320	ENSSSCG00000003348	-	6.4E+07	6.4E+07	6E+07	6E+07	0	6	0.99585	1.3E-09
TCONS_00154320	ENSSSCG00000003343	-	6.4E+07	6.4E+07	6E+07	6E+07	17280	6	0.96343	7.5E-06
TCONS_00154320	ENSSSCG00000038474	*AURKAIP1*	6.4E+07	6.4E+07	6E+07	6E+07	81664	6	0.9874	1.1E-07
TCONS_00154603	ENSSSCG00000003396	*CLSTN1*	7E+07	7E+07	7E+07	7E+07	4637	6	0.97853	9.1E-07
TCONS_00159266	ENSSSCG00000001061	*JARID2*	1.1E+07	1.1E+07	1E+07	1E+07	0	7	0.96678	5.1E-06
TCONS_00160497	ENSSSCG00000001463	*PSMB9*	2.5E+07	2.5E+07	3E+07	3E+07	73751	7	0.95057	2.5E-05
TCONS_00160748	ENSSSCG00000001537	*ZNF76*	3.1E+07	3.1E+07	3E+07	3E+07	45992	7	0.95812	1.3E-05
TCONS_00160748	ENSSSCG00000001539	*PPARD*	3.1E+07	3.1E+07	3E+07	3E+07	0	7	0.9704	3.2E-06
TCONS_00160748	ENSSSCG00000001546	-	3.1E+07	3.1E+07	3E+07	3E+07	50697	7	0.97346	2.1E-06
TCONS_00164862	ENSSSCG00000038886	*CAGE1*	4654450	4655365	5E+06	5E+06	39374	7	0.95158	2.3E-05
TCONS_00165745	ENSSSCG00000024161	-	2.3E+07	2.3E+07	2E+07	2E+07	46583	7	0.98635	1.5E-07
TCONS_00165745	ENSSSCG00000001229	-	2.3E+07	2.3E+07	2E+07	2E+07	60966	7	0.97898	8.3E-07
TCONS_00166048	ENSSSCG00000001456	-	2.5E+07	2.5E+07	2E+07	2E+07	40135	7	0.9853	2E-07
TCONS_00166048	ENSSSCG00000001457	*SLA-DQB1*	2.5E+07	2.5E+07	2E+07	2E+07	28316	7	0.98193	4.6E-07
TCONS_00166048	ENSSSCG00000001455	-	2.5E+07	2.5E+07	2E+07	2E+07	0	7	0.96237	8.4E-06
TCONS_00166048	ENSSSCG00000001458	-	2.5E+07	2.5E+07	2E+07	3E+07	82056	7	0.99246	1.4E-08
TCONS_00166048	ENSSSCG00000001459	*HLA-DOB*	2.5E+07	2.5E+07	2E+07	3E+07	73474	7	0.99193	1.8E-08

TCONS_00166048	ENSSSCG00000031336	-	2.5E+07	2.5E+07	2E+07	2E+07	53862	7	0.98752	1E-07
TCONS_00166770	ENSSSCG00000001638	*MRPS10*	3.7E+07	3.7E+07	4E+07	4E+07	27197	7	0.98732	1.1E-07
TCONS_00166770	ENSSSCG00000001639	*TRERF1*	3.7E+07	3.7E+07	4E+07	4E+07	0	7	0.95248	2.1E-05
TCONS_00169060	ENSSSCG00000002257	*MCTP2*	8.5E+07	8.5E+07	8E+07	8E+07	0	7	0.96243	8.3E-06
TCONS_00174971	ENSSSCG00000008723	*HTRA3*	2882699	2884058	3E+06	3E+06	63004	8	0.95333	2E-05
TCONS_00175874	ENSSSCG00000037950	*NIPAL1*	3.8E+07	3.8E+07	4E+07	4E+07	71054	8	0.97649	1.3E-06
TCONS_00175878	ENSSSCG00000008820	*TEC*	3.8E+07	3.8E+07	4E+07	4E+07	0	8	0.97604	1.4E-06
TCONS_00175878	ENSSSCG00000008821	*SLAIN2*	3.8E+07	3.8E+07	4E+07	4E+07	72351	8	0.97443	1.8E-06
TCONS_00176690	ENSSSCG00000008974	-	7.2E+07	7.2E+07	7E+07	7E+07	35488	8	0.96076	9.9E-06
TCONS_00176844	ENSSSCG00000008989	*CNOT6L*	7.3E+07	7.3E+07	7E+07	7E+07	13957	8	0.95281	2.1E-05
TCONS_00178506	ENSSSCG00000022361	-	1.2E+08	1.2E+08	1E+08	1E+08	29754	8	0.96257	8.2E-06
TCONS_00178703	ENSSSCG00000009211	*PIGY*	1.3E+08	1.3E+08	1E+08	1E+08	20754	8	0.98733	1.1E-07
TCONS_00183321	ENSSSCG00000015433	-	1.1E+08	1.1E+08	1E+08	1E+08	72139	9	0.95578	1.6E-05
TCONS_00183924	ENSSSCG00000015509	*RFWD2*	1.2E+08	1.2E+08	1E+08	1E+08	2282	9	0.9585	1.2E-05
TCONS_00185125	ENSSSCG00000019663	*ssc-mir-29c*	1.3E+08	1.3E+08	1E+08	1E+08	48876	9	0.99402	5.5E-09
TCONS_00185822	ENSSSCG00000018974	*U6*	7463943	7464501	8E+06	8E+06	81038	9	0.97373	2E-06
TCONS_00189240	ENSSSCG00000015410	*PHTF2*	1E+08	1E+08	1E+08	1E+08	0	9	0.98094	5.7E-07
TCONS_00189240	ENSSSCG00000038541	*TMEM60*	1E+08	1E+08	1E+08	1E+08	90169	9	0.98087	5.7E-07
TCONS_00192695	ENSSSCG00000002540	*PPP2R5C*	683422	683804	660880	799618	0	AEMK0200045 2.1	0.95312	0.00002
TCONS_00192695	ENSSSCG00000002541	-	683422	683804	733893	734406	50089	AEMK0200045 2.1	0.954	1.9E-05
TCONS_00192696	ENSSSCG00000002540	*PPP2R5C*	735248	738259	660880	799618	0	AEMK0200045 2.1	0.98792	9.2E-08
TCONS_00192752	ENSSSCG00000002525	*TRAF3*	1529117	1535475	2E+06	2E+06	0	AEMK0200045 2.1	0.97207	2.6E-06

TCONS_00193513	ENSSSCG00000038334	*pRNA*	51110	51310	11403	11488	39622	AEMK0200048<br>9.1	0.95295	2E-05
TCONS_00193951	ENSSSCG00000033114	-	61260	64289	24543	97791	0	AEMK0200052<br>8.1	0.95804	1.3E-05
TCONS_00199048	ENSSSCG00000037591	*AMMECR1*	9E+07	9E+07	9E+07	9E+07	45272	X	0.96052	1E-05

Supplemental Table 5. Loci information of the lncRNA-mRNA pair.

Gene symbol	Chromosome	Seq information	Start Site	End Site	Strand
TCONS_00086451	17	transcript	52,874,827	52,890,259	-
	17	exon	52,873,726	52,874,806	-
	17	exon	52,874,827	52,890,106	-
	17	exon	52,890,191	52,890,259	-
NFATC2	17	transcript	52,743,570	52,890,236	-
	17	exon	52,889,876	52,890,236	-
	17	exon	52,871,515	52,872,517	-
	17	exon	52,866,612	52,866,783	-
	17	exon	52,823,178	52,823,380	-
	17	exon	52,821,785	52,821,957	-
	17	exon	52,807,264	52,807,404	-
	17	exon	52,791,888	52,791,943	-
	17	exon	52,791,358	52,791,484	-
	17	exon	52,788,058	52,788,747	-
	17	exon	52,755,059	52,755,146	-
	17	exon	52,743,570	52,748,034	-

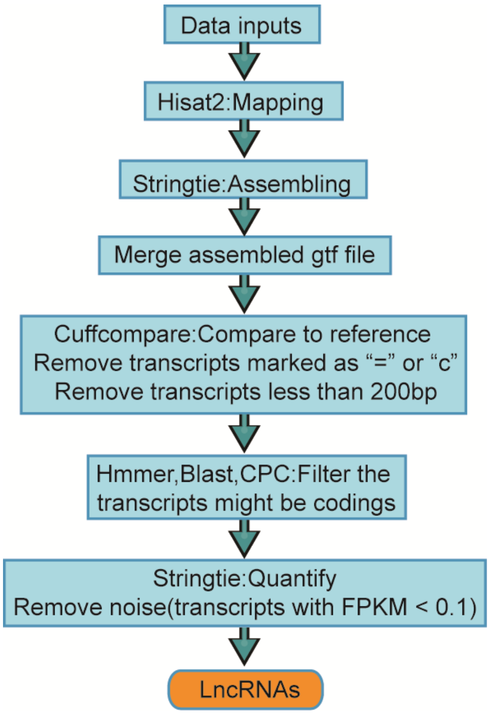

Supplemental Figure S1. LncRNA identification strategy.

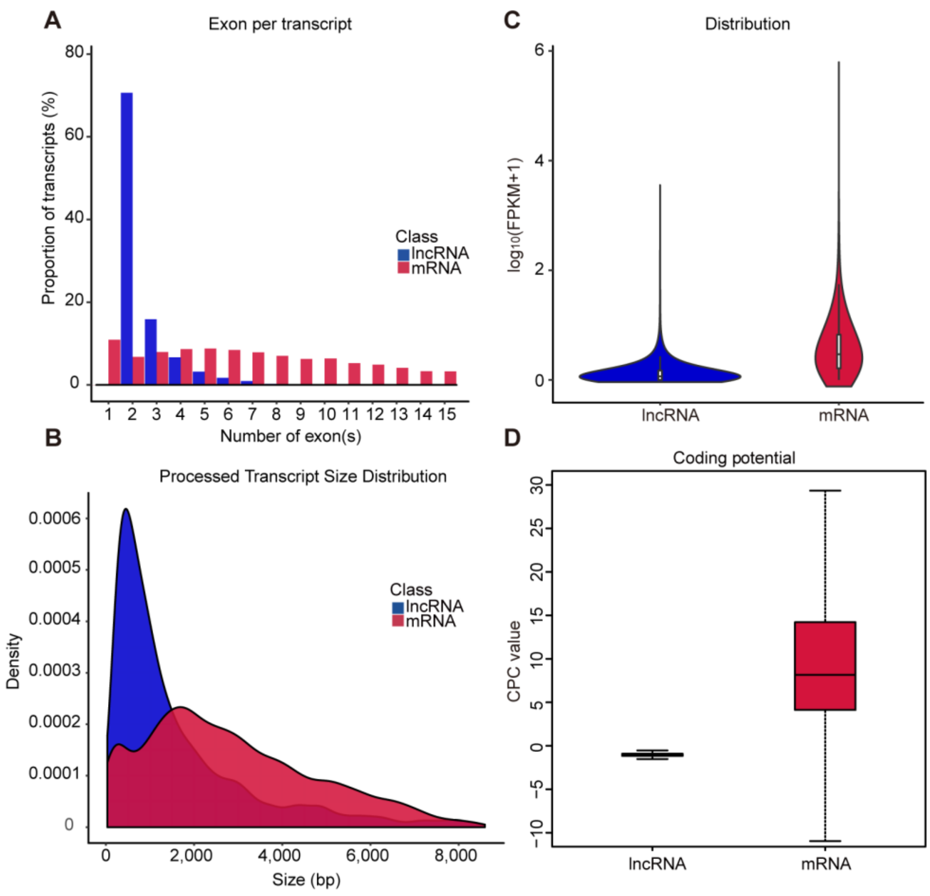

Supplemental Figure S2. Comparison of mRNA and lncRNA characteristics. (A) Exon number, (B) transcripts length, (C) expression level FPKM value transformed by $\log_{10}$ (FPKM+1), and (D) coding potential (CPC (coding potential calculator) was used to calculated CPC value).
